# Long-term safety of COVID vaccination in individuals with idiopathic inflammatory myopathies: results from the COVAD study

**DOI:** 10.1007/s00296-023-05345-y

**Published:** 2023-06-23

**Authors:** Bohdana Doskaliuk, Naveen Ravichandran, Parikshit Sen, Jessica Day, Mrudula Joshi, Arvind Nune, Elena Nikiphorou, Sreoshy Saha, Ai Lyn Tan, Samuel Katsuyuki Shinjo, Nelly Ziade, Tsvetelina Velikova, Marcin Milchert, Kshitij Jagtap, Ioannis Parodis, Abraham Edgar Gracia-Ramos, Lorenzo Cavagna, Masataka Kuwana, Johannes Knitza, Yi Ming Chen, Ashima Makol, Vishwesh Agarwal, Aarat Patel, John D. Pauling, Chris Wincup, Bhupen Barman, Erick Adrian Zamora Tehozol, Jorge Rojas Serrano, Ignacio García-De La Torre, Iris J. Colunga-Pedraza, Javier Merayo-Chalico, Okwara Celestine Chibuzo, Wanruchada Katchamart, Phonpen Akarawatcharangura Goo, Russka Shumnalieva, Leonardo Santos Hoff, Lina El Kibbi, Hussein Halabi, Binit Vaidya, Syahrul Sazliyana Shaharir, A. T. M. Tanveer Hasan, Dzifa Dey, Carlos Enrique Toro Gutiérrez, Carlo V. Caballero-Uribe, James B. Lilleker, Babur Salim, Tamer Gheita, Tulika Chatterjee, Oliver Distler, Miguel A. Saavedra, COVAD study group, Sinan Kardes, Laura Andreoli, Daniele Lini, Karen Schreiber, Melinda Nagy Vince, Yogesh Preet Singh, Rajiv Ranjan, Avinash Jain, Sapan C. Pandya, Rakesh Kumar Pilania, Aman Sharma, Manesh Manoj M, Vikas Gupta, Chengappa G. Kavadichanda, Pradeepta Sekhar Patro, Sajal Ajmani, Sanat Phatak, Rudra Prosad Goswami, Abhra Chandra Chowdhury, Ashish Jacob Mathew, Padnamabha Shenoy, Ajay Asranna, Keerthi Talari Bommakanti, Anuj Shukla, Arunkumar R. Pande, Kunal Chandwar, Akanksha Ghodke, Hiya Boro, Zoha Zahid Fazal, Döndü Üsküdar Cansu, Reşit Yıldırım, Armen Yuri Gasparyan Nicoletta Gianluca Del PapaSambataro, Atzeni Fabiola, Marcello Govoni Simone Parisi, Elena Bartoloni Bocci, Gian Domenico Sebastiani, Enrico Fusaro, Marco Sebastiani Luca Quartuccio, Franco Franceschini, Pier Paolo Sainaghi Giovanni Orsolini, Rossella Maria Giovanna Danielli Vincenzo De AngelisVenerito, Silvia Grignaschi, Alessandro Giollo, Alessia Alluno, Florenzo Ioannone, Marco Fornaro, Lisa S. Traboco, Suryo Anggoro Kusumo Wibowo, Jesús Loarce-Martos, Sergio Prieto-González, Raquel Aranega Gonzalez, Akira Yoshida, Ran Nakashima, Shinji Sato, Naoki Kimura, Yuko Kaneko, Takahisa Gono, Stylianos Tomaras, Fabian Nikolai Proft, Marie-Therese Holzer, Margarita Aleksandrovna Gromova, Or Aharonov, Zoltán Griger, Ihsane Hmamouchi, Imane El bouchti, Zineb Baba, Margherita Giannini, François Maurier, Julien Campagne, Alain Meyer, Daman Langguth, Vidya Limaye, Merrilee Needham, Nilesh Srivastav, Marie Hudson Océane Landon-Cardinal, Wilmer Gerardo Rojas Zuleta, Álvaro Arbeláez Javier Cajas, José António Pereira Silva, João Eurico Fonseca, Olena Zimba Doskaliuk Bohdana, Uyi Ima-Edomwonyi, Ibukunoluwa Dedeke, Emorinken Airenakho, Nwankwo Henry Madu, Abubakar Yerima, Hakeem Olaosebikan, Becky A., Oruma Devi Koussougbo, Elisa Palalane, Ho So, Manuel Francisco Ugarte-Gil, Lyn Chinchay, José Proaño Bernaola, Victorio Pimentel, Hanan Mohammed Fathi, Reem Hamdy A. Mohammed, Ghita Harifi, Yurilís Fuentes-Silva Karoll Cabriza, Jonathan Losanto, Nelly Colaman, Antonio Cachafeiro-Vilar, Generoso Guerra Bautista, Enrique Julio Giraldo Ho, Lilith Stange Nunez, Cristian Vergara M, Jossiell Then Báez, Hugo Alonzo, Carlos Benito Santiago Pastelin, Rodrigo García Salinas, Alejandro Quiñónez Obiols, Nilmo Chávez, Andrea Bran Ordóñez, Gil Alberto Reyes Llerena, Radames Sierra-Zorita, Dina Arrieta, Eduardo Romero Hidalgo, Ricardo Saenz, Idania Escalante M, Wendy Calapaqui, Ivonne Quezada, Gabriela Arredondo, Hector Chinoy, Vikas Agarwal, Rohit Aggarwal, Latika Gupta

**Affiliations:** 1 Department of Pathophysiology, Ivano-Frankivsk National Medical University, Ivano-Frankivsk, Ukraine; 2 Department of Clinical Immunology and Rheumatology, Sanjay Gandhi Postgraduate Institute of Medical Sciences, Lucknow, India; 3 Maulana Azad Medical College, 2-Bahadurshah Zafar Marg, New Delhi, Delhi 110002 India; 4 Department of Rheumatology, Royal Melbourne Hospital, Parkville, VIC 3050 Australia; 5 Walter and Eliza Hall Institute of Medical Research, Parkville, VIC 3052 Australia; 6 Department of Medical Biology, University of Melbourne, Parkville, VIC 3052 Australia; 7 Byramjee Jeejeebhoy Government Medical College and Sassoon General Hospitals, Pune, India; 8 Southport and Ormskirk Hospital NHS Trust, Southport, PR8 6PN UK; 9 Centre for Rheumatic Diseases, King’s College London, London, UK; 10 Rheumatology Department, King’s College Hospital, London, UK; 11 Mymensingh Medical College, Mymensingh, Bangladesh; 12 NIHR Leeds Biomedical Research Centre, Leeds Teaching Hospitals Trust, Leeds, UK; 13 Leeds Institute of Rheumatic and Musculoskeletal Medicine, University of Leeds, Leeds, UK; 14 Division of RheumatologyFaculdade de Medicina FMUSP, Universidade de Sao Paulo, Sao Paulo, SP Brazil; 15 Rheumatology Department, Saint-Joseph University, Beirut, Lebanon; 16 Rheumatology Department, Hotel-Dieu de France Hospital, Beirut, Lebanon; 17 Medical Faculty, Sofia University St. Kliment Ohridski, 1 Kozyak Str, 1407 Sofia, Bulgaria; 18 Department of Internal Medicine, Rheumatology, Diabetology, Geriatrics and Clinical Immunology, Pomeranian Medical University in Szczecin, Ul Unii Lubelskiej 1, 71-252, Szczecin, Poland; 19 Seth Gordhandhas Sunderdas Medical College and King Edwards Memorial Hospital, Mumbai, Maharashtra India; 20 Division of RheumatologyDepartment of Medicine Solna, Karolinska Institutet and Karolinska University Hospital, Stockholm, Sweden; 21 Department of RheumatologyFaculty of Medicine and Health, Örebro University, Örebro, Sweden; 22 Department of Internal Medicine, General Hospital, National Medical Center “La Raza”, Instituto Mexicano del Seguro Social, Av. Jacaranda S/N, Col. La Raza, Del. Azcapotzalco, C.P. 02990 Mexico City, Mexico; 23 Rheumatology UnitDipartimento Di Medicine Interna E Terapia Medica, Università Degli Studi Di Pavia, Pavia, Lombardy Italy; 24 Department of Allergy and Rheumatology, Nippon Medical School Graduate School of Medicine, 1-1-5 Sendagi, Bunkyo-Ku, Tokyo, 113-8602 Japan; 25 Medizinische Klinik 3—Rheumatologie und Immunologie, Universitätsklinikum Erlangen, Friedrich-Alexander-Universität Erlangen-Nürnberg, Ulmenweg 18, 91054 Erlangen, Deutschland; 26 Division of Allergy, Immunology and Rheumatology, Department of Internal Medicine, Taichung Veterans General Hospital, Taichung City, Taiwan; 27 Department of Medical Research, Taichung Veterans General Hospital, Taichung, Taiwan; 28 Division of Rheumatology, Mayo Clinic, Rochester, MN USA; 29 Mahatma Gandhi Mission Medical College, Navi Mumbai, Maharashtra India; 30 Bon Secours Rheumatology Center and Division of Pediatric RheumatologyDepartment of Pediatrics, University of Virginia School of Medicine, Charlottesville, VA USA; 31 Bristol Medical School Translational Health Sciences, University of Bristol, Bristol, UK; 32 Department of Rheumatology, North Bristol NHS Trust, Bristol, UK; 33 Division of Medicine, Rayne InstituteDepartment of Rheumatology, University College London, London, UK; 34 Centre for Adolescent Rheumatology Versus Arthritis at UCL, UCLH, GOSH, London, UK; 35 Department of General Medicine, All India Institute of Medical Sciences (AIIMS), Guwahati, India; 36 Rheumatology, Medical Care & Research, Centro Medico Pensiones Hospital, Instituto Mexicano del Seguro Social Delegación Yucatán, Yucatán, Mexico; 37 Rheumatologist and Clinical InvestigatorInterstitial Lung Disease and Rheumatology Unit, Instituto Nacional de Enfermedades Respiratorias, Mexico City, Mexico; 38 Departamento de Inmunología Y Reumatología, Hospital General de Occidente and Universidad de Guadalajara, Guadalajara, Jalisco Mexico; 39 Hospital Universitario Dr Jose Eleuterio Gonzalez, Monterrey, Mexico; 40 Department of Immunology and Rheumatology, Instituto Nacional de Ciencias Médicas Y Nutrición Salvador Zubirán, Mexico City, Mexico; 41 Department of Medicine, University of Nigeria Teaching Hospital, Ituku-Ozalla/University of Nigeria, Enugu Campus, Enugu, Nigeria; 42 Division of RheumatologyDepartment of MedicineFaculty of Medicine Siriraj Hospital, Mahidol University, Bangkok, Thailand; 43 Department of Medicine, Queen Savang Vadhana Memorial Hospital, Chonburi, Thailand; 44 Department of RheumatologyClinic of Rheumatology, University Hospital “St. Ivan Rilski”, Medical University-Sofia, Sofia, Bulgaria; 45 School of Medicine, Universidade Potiguar (UnP), Natal, Brazil; 46 Internal Medicine Department, Rheumatology Unit, Specialized Medical Center, Riyadh, Saudi Arabia; 47 Department of Internal MedicineSection of Rheumatology, King Faisal Specialist Hospital and Research Center, Jeddah, Saudi Arabia; 48 National Center for Rheumatic Diseases (NCRD), Ratopul, Kathmandu Nepal; 49 Faculty of Medicine, Universiti Kebangsaan Malaysia, 56000 Cheras, Kuala Lumpur Malaysia; 50 Department of Rheumatology, Enam Medical College & Hospital, Dhaka, Bangladesh; 51 Department of Medicine and Therapeutics, Rheumatology Unit, University of Ghana Medical SchoolCollege of Health Sciences, Korle-Bu, Accra, Ghana; 52 Reference Center for Osteoporosis, Rheumatology and Dermatology, Pontifica Universidad Javeriana Cali, Cali, Colombia; 53 Department of Medicine, Hospital Universidad del Norte, Barranquilla, Atlantico Colombia; 54 Division of Musculoskeletal and Dermatological Sciences, Centre for Musculoskeletal Research, School of Biological Sciences, Faculty of Biology, Medicine and Health, Manchester Academic Health Science Centre, The University of Manchester, Manchester, UK; 55 Manchester Centre for Clinical Neurosciences, Salford Royal NHS Foundation Trust, Salford, UK; 56 Rheumatology Department, Fauji Foundation Hospital, Rawalpindi, Pakistan; 57 Rheumatology Department, Kasr Al Ainy School of Medicine, Cairo University, Cairo, Egypt; 58 Department of Internal Medicine, University of Illinois College of Medicine at Peoria, Peoria, IL USA; 59 Department of Rheumatology, University Hospital Zurich, University of Zurich, Zurich, Switzerland; 60 Departamento de Reumatología Hospital de Especialidades Dr. Antonio Fraga Mouret, Centro Médico Nacional La Raza, IMSS, Mexico City, Mexico; 61 National Institute for Health Research Manchester Biomedical Research Centre, Manchester University NHS Foundation Trust, The University of Manchester, Manchester, UK; 62 Department of Rheumatology, Salford Royal Hospital, Northern Care Alliance NHS Foundation Trust, Salford, UK; 63 Division of Rheumatology and Clinical Immunology, University of Pittsburgh School of Medicine, Pittsburgh, PA USA; 64 Department of Rheumatology, Royal Wolverhampton Hospitals NHS Trust, Wolverhampton, UK; 65 City Hospital, Sandwell and West Birmingham Hospitals NHS Trust, Birmingham, UK

**Keywords:** COVID-19, Vaccination, Adverse event, Myositis, Autoimmunity, Surveys and questionnaires

## Abstract

Limited evidence on long-term COVID-19 vaccine safety in patients with idiopathic inflammatory myopathies (IIMs) continues to contribute to vaccine hesitancy. We studied delayed-onset vaccine adverse events (AEs) in patients with IIMs, other systemic autoimmune and inflammatory disorders (SAIDs), and healthy controls (HCs), using data from the second COVID-19 Vaccination in Autoimmune Diseases (COVAD) study. A validated self-reporting e-survey was circulated by the COVAD study group (157 collaborators, 106 countries) from Feb–June 2022. We collected data on demographics, comorbidities, IIM/SAID details, COVID-19 history, and vaccination details. Delayed-onset (> 7 day) AEs were analyzed using regression models. A total of 15165 respondents undertook the survey, of whom 8759 responses from vaccinated individuals [median age 46 (35–58) years, 74.4% females, 45.4% Caucasians] were analyzed. Of these, 1390 (15.9%) had IIMs, 50.6% other SAIDs, and 33.5% HCs. Among IIMs, 16.3% and 10.2% patients reported minor and major AEs, respectively, and 0.72% (*n* = 10) required hospitalization. Notably patients with IIMs experienced fewer minor AEs than other SAIDs, though rashes were expectedly more than HCs [OR 4.0; 95% CI 2.2–7.0, *p* < 0.001]. IIM patients with active disease, overlap myositis, autoimmune comorbidities, and ChadOx1 nCOV-19 (Oxford/AstraZeneca) recipients reported AEs more often, while those with inclusion body myositis, and BNT162b2 (Pfizer) recipients reported fewer AEs. Vaccination is reassuringly safe in individuals with IIMs, with AEs, hospitalizations comparable to SAIDs, and largely limited to those with autoimmune multimorbidity and active disease. These observations may inform guidelines to identify high-risk patients warranting close monitoring in the post-vaccination period.

**Supplementary Information:**

The online version contains supplementary material available at 10.1007/s00296-023-05345-y.

## Introduction

Vaccination has been one of the most effective measures in reducing the mortality and severe outcomes of COVID-19, significantly reducing the burden on the healthcare infrastructure [1]. However, it is concerning to note occasional reports of delayed adverse events (AEs), including exacerbation of underlying systemic autoimmune diseases (SAIDs), and even de novo induction of SAIDs associated with vaccination, as it is progressively introduced in various patients groups [2–6].

Individuals living with idiopathic inflammatory myopathies (IIMs), many of whom receive disease modifying drugs (DMARDs) and glucocorticoids, are particularly vulnerable to severe COVID-19 outcomes, and thus improving vaccine uptake in this group may limit these severe outcomes [7]. However, owing to the rare nature of this disease, patients with IIM are scarcely represented, with only a few large-scale studies exploring the safety, tolerability, and immunogenicity of COVID-19 vaccines in this group [8, 9]. The first COVID-19 Vaccination in Autoimmune Diseases (COVAD) study established the short-term 7-day vaccine safety, with AEs being comparable between patients with IIMs, other SAIDs, and health controls (HCs). Most of the events were limited to individuals with active disease and autoimmune multimorbidity, a group already predisposed to high background prevalence of rashes while individuals with inclusion body myositis (IBM) reported fewer events [9, 10]. While the short-term safety of vaccines is well characterized, a considerable gap exists in our understanding of the delayed effects of vaccination in this vulnerable group, owing to a lack of follow-up prospective studies evaluating delayed-onset AEs.

This is a critical issue, potentially contributing to persisting vaccine hesitancy among these patients. Recent analysis from the second COVAD study revealed concerns over long-term vaccine safety had increased among patients with IIMs and SAIDs, and remained a significant cause of hesitancy [11]. This warrant concern, being an impediment achieving herd immunity in this high-risk group. Interestingly, this pattern of hesitancy is not seen in response to other major inoculation campaigns such as influenza [12]. Thus, we may infer that the hesitancy to COVID-19 vaccination in this patient group may not stem majorly from general antivaccination sentiments, but rather in response to specific concerns regarding COVID-19 vaccines. Indeed, the lack of reliable information regarding the possible deterioration of disease course and development of AEs may lead to misinformation, and precipitate this hesitancy [13]. Thus, the further identification and analysis of possible delayed-onset and long-term AEs of COVID-29 vaccination represent an urgent and largely unmet need, being essential to providing evidence-based information to reduce hesitancy and improve vaccination coverage in this patient group. Therefore, we analyzed the delayed-onset (> 7 day) AEs of COVID-19 vaccination in patients with IIMs, other SAIDs, and HCs, using data from the second international COVAD patient self-reported multi-center e-survey [14].

## Methods

### Study design

This study was conducted as part of the second COVAD study, an ongoing cross-sectional, multi-center patient self-reported online survey [14]. Participants consented electronically after being informed via a cover letter in lieu of written consent, and approval was obtained from the local institutional ethics committee, we adhered to the Checklist for Reporting Results of the Internet E-Surveys (CHERRIES) [15, 16].

### Data collection

A validated questionnaire was hosted on the surveymonkey.com online platform, following pilot testing, vetting, and revision by an international team of experts, and translation into 18 languages, and was circulated extensively by the COVAD study group of 157 collaborators across 106 countries in their clinics, patient support groups, and social media platforms from February to June 2022 [14].

We collected data on demographic details, comorbidities, SAID diagnosis, treatment details, current symptom status, COVID-19 infection history, course, and outcomes (including hospitalization and need for oxygen therapy), COVID-19 vaccination details, short-term (< 7 day) and delayed-onset (> 7 day) post-vaccination AEs (based on CDC criteria), and patient-reported outcomes as per the Patient Reported Outcomes Measurement Information System (PROMIS) [17]. All individuals over the age of 18 years, including patients with multiple overlapping autoimmune diseases were included in this study. Duplication of responses from a single respondent was averted due to the electronic protocols. Methods have been previously detailed at length in the available COVAD study protocol [14].

### Data extraction

**Fig. 1:**
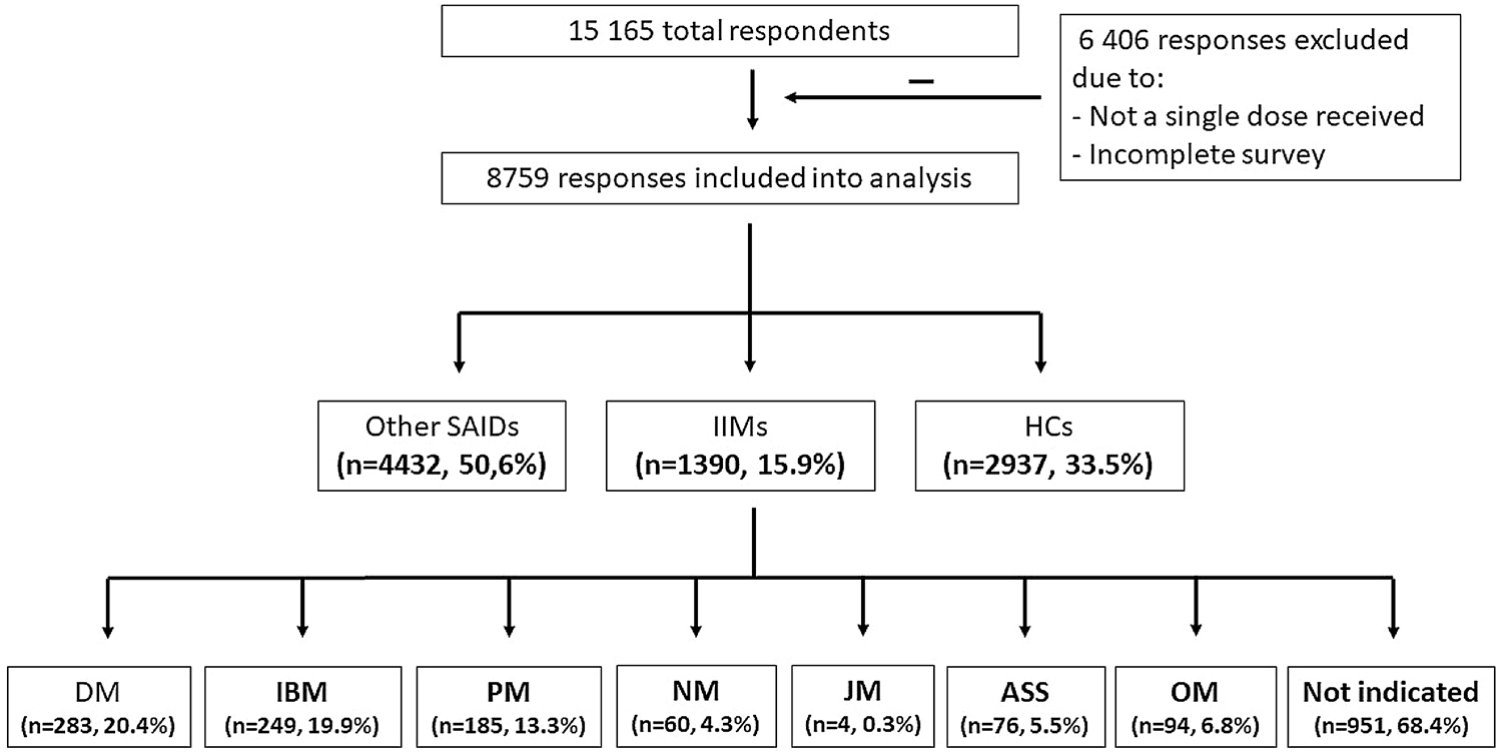
Flow diagram of data extraction

Data were extracted on 10th July 2022. Only responses from respondents who completed the survey in full and had received at least one dose of any COVID-19 vaccine at the time of survey completion were included in the analysis (Fig. 1). Variables extracted included relevant outcome measures, delayed-onset self-reported vaccine AEs, as well as baseline socio-demographic and clinical characteristics, and vaccination status.

### Active and inactive disease

Patients self-reported their disease activity as “inactive/remission”, “active and improving”, active but stable”, “active and worsening”, “I am not sure”, or “other”. Disease status was additionally verified based on reported symptom status and treatment regime prior to vaccination.

### Adverse events post-vaccination

Delayed-onset ADEs were those occurring > 7 days post-vaccination, and were categorized into minor AEs, major AEs requiring urgent medical attention (but not hospitalization), and hospitalizations [18]. Survey participants were able to report additional not listed AEs as “others” via an open-ended question.

### Statistical analysis

The type of data distribution was determined by Kolmogorov–Smirnov and Shapiro–Wilk tests. The continuous variables were distributed non-parametrically. Thus, descriptive statistics were represented as median (IQR). For analyzing the statistical difference between categorical and continuous variables, Chi-square and Mann–Whitney-*U* tests were used, respectively. Fisher test was applied to compare categorical data in case of variable frequency count less than 5. We compared differences in AEs between IIMs, SAIDs, and HCs with sub-group analysis by subtype of IIMs, vaccine received, disease activity, autoimmune and non-autoimmune comorbidities (in myositis patients), and immunosuppressive therapy received.

The variables that were found significant in univariable analysis, and those suspected of being clinically important, were further evaluated in binary logistic regression analysis (BLR) with adjustment for factors deemed relevant based on evidence from current literature and clinical judgment, including age, gender, ethnicity, comorbidity, immunosuppressive therapy, number and type of vaccines received and stratified by country of origin by Human Development Index (HDI) (which served as a surrogate marker for socioeconomic status) [19]. *p* < 0.05 was considered significant. Statistical analysis was carried out using IBM SPSS version 26.

## Results

### Baseline characteristics

A total of 15165 respondents undertook the survey, of whom complete responses from 8759 vaccinated respondents were included in the analysis (Fig. 1). The included participants had a median age of 46 (35–58) years, were mostly female (74.4%) and Caucasians (45.4%), with 1390 (15.9%) having IIMs, while 50.6% had other SAIDs, and 33.5% were HCs. In addition to IIMs, the most frequent SAIDs in our sample were rheumatoid arthritis (18.8%) and Sjogren’s syndrome (12.6%). Nearly all (97%) respondents received two COVID-19 vaccine doses, and 15.8% received four doses, with the majority of vaccine uptake contributed by the BNT162b2 (Pfizer)-BioNTech (61.1%) and the ChadOx1 nCOV-19 (Oxford/AstraZeneca) (29.4%) vaccines.

Among patients with IIMs, the dermatomyositis sub-group was predominant (20.4%), followed by inclusion body myositis (17.9%) and polymyositis (13.3%). Other socio-demographic and clinical characteristics are detailed in Table 1, and Supplementary Tables 1, 2, 3, and 9.

**Table 1:** Socio-demographic and basic clinical features of the survey respondents

Variable	Total, *n* (%) 8759 (100)	IIM, *n* (%) 1390 (15.87)	SAIDs, *n* (%) 4432 (50.60)	HC, *n* (%) 2937 (33.53)
Age (median, IQR), years	46 (35–58)	62 (50–71)	47 (36–58)	38 (29–49)
Gender F:M	6518:2189 (2.98:1)	990:393 (2.52:1)	3735:670 (5.57:1)	1788:1126 (1.59:1)
Pregnancy (positive status), *n* (%)	62 (0.7)	5 (0.4)	28 (0.6)	29 (1.0)
Lactating/breastfeeding (positive status), * n* (%)	118 (1.3)	11 (0.8)	57 (1.3)	50 (1.7)
Ethnicity, * n* (%)				
African American or of African origin (Black)	376 (4.3)	56 (4.0)	255 (5.8)	65 (2.2)
Asian	1843 (21.0)	97 (7.0)	984 (22.2)	762 (25.9)
Caucasian (White)	3980 (45.4)	1113 (80.1)	2054 (46.3)	813 (27.7)
Do not wish to disclose	293 (3,3)	19 (1.4)	153 (3.5)	121 (4.1)
Hispanic	1481 (16.9)	55 (4.0)	579 (13.1)	847 (28.8)
Native American/Indigenous/Pacific Islander	69 (0.8)	5 (0.4)	38 (0.9)	26 (0.9)
Other	717 (8.2)	45 (3.2)	369 (8.3)	303 (10.3)
Vaccines, * n* (%)				
BNT162b2 (Pfizer)-BioNTech	5354 (61.1)	883 (63.5)	2939 (66.3)	1532 (52.2)
ChadOx1 nCOV-19 (Oxford/AstraZeneca)	2579 (29.4)	175 (12.6)	1507 (34.0)	897 (30.5)
JNJ-78436735 (Johnson and Johnson)	268 (3.1)	53 (3.8)	111 (2.5)	104 (3.5)
MRNA-1273 (Moderna)	1880 (21.5)	555 (39.9)	883 (19.9)	442 (15)
NVX-CoV2373 (Novovax)	22 (0.3)	5 (0.4)	8 (0.2)	9 (0.3)
ChAdOx1 nCoV-19 (Covishield Serum Institute India)	510 (5.8)	15 (1.1)	214 (4.8)	281 (9.6)
BBV152 (Covaxin Bharat Biotech)	81 (0.9)	7 (0.5)	34 (0.8)	40 (1.4)
Gam-COVID-Vac (Sputnik)	331 (3.8)	6 (0.4)	113 (2.5)	212 (7.2)
BBIBP-CorV (Sinopharm)	579 (6.6)	24 (1.7)	243 (5.5)	312 (10.6)
Sinovac-CoronaVac	695 (7.9)	29 (2.1)	361 (8.1)	305 (10.4)
Not sure	117 (1.3)	6 (0.4)	57 (1.3)	54 (1.8)
Minor ADEs duration, median (IQR), days	5 (2–10)	6 (3–13.3)	6 (3–13)	4 (2–7)
Major ADEs duration, median (IQR), days	8 (3–35)	17 (5–90)	9 (3–35.5)	6 (2–17)

*HC* healthy control, *IIM* idiopathic inflammatory myopathy, *SAID* systemic autoimmune and inflammatory disease

The country of origin of the respondents is detailed in Supplementary Table 11.

### Post-COVID-19 vaccination-associated AEs in patients with IIM compared to SAIDs and HCs

Among patients with IIMs, any minor delayed-onset AEs were seen in 16.3% respondents, while major AEs were reported by 10.2%. Fatigue (8.8%) and local injection site (arm) pain/ soreness (8.3%) were the most commonly reported minor AEs, while among major AEs, difficulty in breathing (3.3%) was most frequent. Reassuringly, hospitalizations associated with COVID-19 vaccination were rare in patients with IIMs (0.72%).

Notably patients with IIMs were at a lower risk of local injection site pain [OR 0.8 (0.6–1.0. *p* = 0.030]. joint pain [OR 0.6 (0.5–0.8), *p* < 0.001], headache [OR 0.6 (0.5–0.9), *p* = 0.002], fatigue [OR 0.7 (0.6–0.9)], *p* = 0.014], and dizziness [OR 0.7 (0.5–0.9), *p* = 0.024] than SAIDs (Suppl. Table 5). We noted with concern that patients with IIMs had a higher risk of development of both mild and severe rases than HCs [OR 4.0 (2.2–7.0), *p* < 0.001 and OR 2.1 (1.2–3.5), *p* = 0.006 respectively], though reassuringly this increased risk was lost when the effect of immunosuppressive therapy was adjusted for in BLR suggesting possible underlying confounding effect of active disease (Suppl. Table 5).

AEs appeared relatively later among IIMs compared to SAIDs and HCs, with a longer post-vaccination median duration to appearance of AEs [17 (5–90) days in IIMs vs. 9 (3–3.5) days in SAIDs and 6 (2–17) days in HCs] (Table 2).

**Table 2:** Effects of COVID-19 vaccination in patients with IIMs vs. other SAIDs and HCs

	IIM		SAIDs		HCs		OR1 (95%CI)	OR2 (95%CI)	p1	p2
	*N* (1390)	% (100)	*N* (4432)	% (100)	*N* (2937)	% (100)				
**Minor AEs**	227	16.3	948	21.4	561	19.1	0.7 (0.6–0.8)	0.8 (0.7–1.0)	< .001	0.027
Injection site (arm) pain and soreness	115	8.3	558	12.6	365	12.4	0.6 (0.5–0.8)#	0.6 (0.5–0.8)	< .001	< .001
Myalgia	103	7.4	443	10.0	217	7.4	0.7 (0.6–0.9)		0.004	0.980
Body ache	108	7.8	488	11.0	238	8.1	0.7 (0.5–0.8)		0.001	0.705
Joint pain	91	6.5	486	11.0	165	5.6	0.6 (0.5–0.7)#		< .001	0.227
Fever	71	5.1	359	8.1	248	8.4	0.6 (0.5–0.8)	0.6 (0.4–0.8)	< .001	< .001
Chills	72	5.2	285	6.4	162	5.5			0.09	0.648
Cough	23	1.7	111	2.5	54	1.8			0.065	0.669
Difficulty in breathing or shortness of breath	36	2.6	126	2.8	58	2.0			0.617	0.195
Nausea/vomiting	29	2.1	171	3.9	45	1.5	0.5 (0.4–0.8)		0.002	0.189
Headache	86	6.2	428	9.7	193	6.6	0.6 (0.5–0.8)#		< .001	0.631
Rash	55	4.0	129	2.9	27	0.9		4.4 (2.8–7.1)	0.052	< .001
Fatigue	122	8.8	507	11.4	198	6.7	0.7 (0.6–0.9)#	1.3 (1.1–1.7)	0.005	0.017
Diarrhea	25	1.8	117	2.6	42	1.4			0.076	0.359
Abdominal pain	24	1.7	101	2.3	33	1.1			0.215	0.104
High pulse rate or palpitations	36	2.6	167	3.8	73	2.5	0.7 (0.5–1.0)		0.037	0.838
Rise in blood pressure	19	1.4	86	1.9	30	1.0			0.161	0.316
Fainting	4	0.3	22	0.5	12	0.4			0.309	0.541
Dizziness	43	3.1	221	5.0	68	2.3	0.6 (0.4–0.8)#		0.003	0.131
Chest pain	16	1.2	120	2.7	30	1.0	0.4 (0.2–0.7)		0.001	0.698
Swelling in the extremities	21	1.5	100	2.3	29	1.0			0.089	0.133
Weakness and tingling in the feet and legs	47	3.4	166	3.7	65	2.2		1.5 (1.1–2.3)	0.528	0.024
Pricking or pins and needles sensations in the hands and feet	36	2.6	137	3.1	42	1.4		1.8 (1.2–2.9)	0.337	0.007
Visual disturbances (loss of vision, blurring of vision, etc.)	17	1.2	115	2.6	28	1.0			0.003	0.414
Bleeding/bruising on the body	14	1.0	67	1.5	15	0.5			0.161	0.062
Petechial rash	11	0.8	54	1.2	11	0.4			0.186	0.072
**Major AEs**	142	10.2	685	15.5	375	12.8	0.6 (0.5–0.8)	0.8 (0.6–1.0)	< 0.001	0.016
Anaphylaxis	20	1.4	66	1.5	47	1.6			0.892	0.688
Marked difficulty in breathing	46	3.3	135	3.0	77	2.6			0.622	0.204
Throat closure	24	1.7	63	1.4	38	1.3			0.413	0.263
Severe rashes	42	3.0	108	2.4	54	1.8		1.7 (1.1–2.5)	0.23	0.014
**Hospitalization**	41	2.9	201	4.5	77	2.6	0.6 (0.5–0.9)		0.01	0.536

*AE* adverse event, *CI* confidence interval, *HC* healthy control, *IIM* idiopathic inflammatory myopathy, *OR* odds ratio, *SAID* systemic autoimmune and inflammatory disease

^#^Significant in BLR (binary logistic regression) adjusted for age, gender, ethnicity, immunosuppressant dose, and stratified by country

OR 1 and 2 compares AEs between IIM and SAIDs, and IIM and HCs, respectively

### Post-COVID-19 vaccination-associated AEs in patients across different IIM subtypes

Among patients with IIMs, those with overlap myositis (OM) had the highest absolute risk of minor [OR 4.4 (2.8–6.9), *p* < 0.001] and major AEs [OR 4.1 (2.4–7.1), *p* < 0.001] compared to other subtypes of IIMs (Table 3, Suppl. Table 5). Patients with OM were also at a higher risk of hospitalization [8.5% vs. 0–5%; OR 3.9 (1.4–11.0), *p* = 0.011], though reassuringly with small absolute numbers (3–10) across all subtypes. Conversely, patients with IBM patients were relatively protected from AEs, having a lower risk of myalgia, joint pain, and rash (Suppl. Table 5).

**Table 3:** COVID-19 vaccination-associated AEs across different IIM subtypes

	DM (283)		IBM (249)		PM (185)		NM (60)		JM (4)		ASS (76)		OM (94)	
	*N*	%	*N*	%	*N*	%	*N*	%	*N*	%	*N*	%	*N*	%
**Minor AEs**	47	16.6	18***#	7.2	38	20.5	12	20	1	25.0	11	14.5	41***#	43.6
Injection site (arm) pain and soreness	26	9.2	8***	3.2	17	9.2	9	15.0	0	0.0	6	7.9	17**#	18.1
Myalgia	23	8.1	5***#	2.0	18	9.7	4	6.7	0	0.0	5	6.6	18***#	19.1
Body ache	27	9.5	6***	2.4	13	7.0	3	5.0	0	0.0	3	3.9	24***#	25.5
Joint pain	22	7.8	3***#	1.2	10	5.4	3	5.0	0	0.0	5	6.6	18***#	19.1
Fever	16	5.7	4*	1.6	6	3.2	1	1.7	0	0.0	4	5.3	14***#	14.9
Chills	20*	7.1	4*	1.6	4	2.2	1	1.7	0	0.0	4	5.3	13***#	13.8
Cough	5	1.8	0*	0.0	0	0.0	0	0.0	1***	25.0	1	1.3	6***#	6.4
Difficulty in breathing or shortness of breath	10	3.5	2*	0.8	4	2.2	1	1.7	0	0.0	1	1.3	8***#	8.5
Nausea/vomiting	8	2.8	1*	0.4	1	0.5	1	1.7	0	0.0	1	1.3	7***#	7.4
Headache	19	6.7	4***	1.6	13	7.0	3	5.0	0	0.0	3	3.9	18***#	19.1
Rash	16	5.7	1**#	0.4	8	4.3	0	0.0	0	0.0	1	1.3	11***#	11.7
Fatigue	29	10.2	9***	3.6	14	7.6	5	8.3	0	0.0	7	9.2	23***#	24.5
Diarrhea	7	2.5	0*	0.0	2	1.1	1	1.7	0	0.0	0	0.0	5**#	5.3
Abdominal pain	6	2.1	1	0.4	1	0.5	1	1.7	0	0.0	1	1.3	5**#	5.3
High pulse rate or palpitations	10	3.5	1*	0.4	1	0.5	0	0.0	0	0.0	3	3.9	9***#	9.6
Rise in blood pressure	4	1.4	2	0.8	2	1.1	0	0.0	0	0.0	1	1.3	3	3.2
Fainting	1	0.4	0	0.0	0	0.0	0	0.0	0	0.0	0	0.0	1	1.1
Dizziness	10	3.5	3	1.2	5	2.7	0	0.0	0	0.0	2	2.6	8**#	8.5
Chest pain	5*	1.8	1	0.4	1	0.5	0	0.0	0	0.0	0	0.0	1	1.1
Swelling in the extremities	3	1.1	1	0.4	1	0.5	0	0.0	0	0.0	2*	2.6	0	0.0
Weakness and tingling in the feet and legs	6	2.1	1*	0.4	12**	6.5	1	1.7	0	0.0	0	0.0	8**#	8.5
Pricking or pins and needles sensations in the hands and feet	3	1.1	1*	0.4	7	3.8	1	1.7	0	0.0	1	1.3	7***#	7.4
Visual disturbances (loss of vision, blurring of vision, etc.)	4	1.4	1	0.4	3	1.6	0	0.0	0	0.0	0	0.0	3	3.2
Bleeding/bruising on the body	1	0.4	1	0.4	2	1.1	0	0.0	0	0.0	0	0.0	1	1.1
Petechial rash	2	0.7	0	0.0	0	0.0	0	0.0	0	0.0	0	0.0	4***#	4.3
**Major AEs**	24	8.5	13*#	5.2	17	9.2	6	10	1	25.0	3	3.9	23***#	24.5
Anaphylaxis	4	1.4	3	1.2	2	1.1	0	0.0	0	0.0	0	0.0	3	3.2
Marked difficulty in breathing	11	3.9	4	1.6	7	3.8	1	1.7	1*	25.0	1	1.3	7*	7.4
Throat closure	5	1.8	4	1.6	3	1.6	0	0.0	0	0.0	0	0.0	4*	4.3
Severe rashes	11	3.9	5	2.0	4	2.2	0	0.0	0	0.0	0	0.0	7*#	7.4
**Hospitalization**	7	2.5	4	1.6	4	2.2	3	5.0	0	0.0	1	1.3	8***#	8.5

Comparisons are between each IIM subtype vs. the rest of IIM subtypes. Bold indicates increased OR vs. the others. Bold + Underlined indicates decreased OR vs. the others. *AE* adverse events, *ASS* anti-synthetase syndrome, *DM* dermatomyositis, *IBM* inclusion body myositis, *IIM* idiopathic inflammatory myopathies, *JDM* juvenile dermatomyositis, *NM* necrotizing myositis, *OM* overlap myositis, *PM* polymyositis

^#^Significant in BLR (binary logistic regression) adjusted for age, gender, ethnicity, immunosuppressant dose, and stratified by country. *p < .05, **p < .005, ***p < .001

### Comparison of post-COVID-19 vaccination AE among IIM patients by vaccine type

Patients with IIMs who received the BNT162b2 (Pfizer) vaccine were at a lower risk of injection site pain/soreness [OR 0.6 (0.4–1.0), *p* = 0.039], petechial rash [OR 0.2 (0.04–0.8), *p* = 0.026], and certain other minor AEs compared to other vaccines (Table 4, Suppl. Table 6a). Post-vaccination flares of underlying autoimmune disease were also less frequent among IIMs than SAIDs [OR 0.8 (0.6–1.0), *p* = 0.032]. However, we noted with concern that among BNT162b2 (Pfizer) vaccine recipients, patients with IIMs were at a threefold higher risk of rash compared to HCs [OR 3.0 (1.4–6.3), *p* = 0.004].

**Table 4:** AEs distribution according to the vaccines in IIM group

	BNT162b2 (Pfizer)		ChadOx1 nCOV-19 (Oxford/ AstraZeneca)		mRNA-1273 (Moderna)		ChAdOx1 nCoV-19 (Covishield Serum Institute India)		Sinovac-CoronaVac	
	*N* (883)	% (100)	*N* (175)	% (100)	*N* (555)	% (100)	*N* (15)	% (100)	*N* (29)	% (100)
**Minor AEs**	136	15.4	38*	21.7	84	15.1	4	26.7	9*	31.0
Injection site (arm) pain and soreness	65*#	7.4	22	12.6	10	1.8	1	6.7	6*	20.7
Myalgia	59	6.7	18	10.3	8	1.4	1	6.7	5*	17.2
Body ache	59*	6.7	20	11.4	8	1.4	2	13.3	6*	20.7
Joint pain	52	5.9	17	9.7	8	1.4	2	13.3	5*#	17.2
Fever	41	4.6	15	8.6	5	0.9	2	13.3	5*	17.2
Chills	44	5.0	13	7.4	6	1.1	1	6.7	3	10.3
Cough	13	1.5	1	0.6	1	0.2	1	6.7	3***#	10.3
Difficulty in breathing or shortness of breath	22	2.5	4	2.3	4	0.7	2*	13.3	2	6.9
Nausea/vomiting	13	1.5	5	2.9	1	0.2	0	0.0	0	0.0
Headache	55	6.2	17*#	9.7	5	0.9	0	0.0	3	10.3
Rash	24**#**	2.7	3	1.7	4	0.7	3***#	20.0	2	6.9
Fatigue	71	8.0	21	12.0	6	1.1	2	13.3	4	13.8
Diarrhea	16	1.8	6	3.4	2	0.4	1	6.7	1	3.4
Abdominal pain	10	1.1	4	2.3	2	0.4	1	6.7	3***#	10.3
High pulse rate or palpitations	25	2.8	5	2.9	1	0.2	2*	13.3	4***#	13.8
Rise in blood pressure	13	1.5	6*#	3.4	1	0.2	0	0.0	1	3.4
Fainting	2	0.2	2	1.1	1	0.2	1**	6.7	2***#	6.9
Dizziness	29	3.3	8	4.6	2	0.4	1	6.7	4**#	13.8
Chest pain	10	1.1	3	1.7	1	0.2	0	0.0	1	3.4
Swelling in the extremities	13	1.5	6	3.4	2	0.4	1	6.7	2*	6.9
Weakness and tingling in the feet and legs	27	3.1	10	5.7	2	0.4	1	6.7	4**#	13.8
Pricking or pins and needles sensations in the hands and feet	21	2.4	8	4.6	1	0.2	1	6.7	5***#	17.2
Visual disturbances (loss of vision, blurring of vision, etc.)	10	1.1	5	2.9	2	0.4	1	6.7	2*#	6.9
Bleeding/bruising on the body	10	1.1	6*#	3.4	1*	0.2	1	6.7	2*	6.9
Petechial rash	3*#	0.3	2	1.1	1	0.2	1*	6.7	2***	6.9
**Major AEs**	72**#	8.2	22	12.6	61	11.0	7***#	46.7	10***#	34.5
Anaphylaxis	11*	1.2	5	2.9	9	1.6	3***#	20.0	5***#	17.2
Marked difficulty in breathing	26	2.9	7	4.0	10	1.8	5***#	33.3	4**	13.8
Throat closure	13**	1.5	6	3.4	9	1.6	3***#	20.0	5***#	17.2
Severe rashes	19**#	2.2	6	3.4	10	1.8	4***#	26.7	6***#	20.7
**Hospitalization**	21**	2.4	8	4.6	11	2.0	4***#	26.7	5***#	17.2

Bold indicates increased odds ratio vs. the remaining vaccines. Bold + Underlined indicates decreased odds ratio vs. remaining vaccines. *AE* adverse events, *AID* autoimmune disease, *HC* healthy control, *IIM* idiopathic inflammatory myopathy

^#^Significant according to binary logistic regression adjusted for age, gender, ethnicity, and immunosuppressant dose, and stratified by country. *p < .05, **p < .005, ***p < .001

ChadOx1 nCOV-19 (Oxford/ AstraZeneca) vaccine recipients were more prone to develop bleeding/bruising on the body [OR 6.8 (2.0–22.9), *p* = 0.007] compared to other vaccines albeit with wide confidence intervals. The risk of post-vaccination headache and rise in blood pressure was also higher (Suppl. Table 6d).

We found myositis patients receiving the Sinovac-CoronaVac and ChAdOx1 nCoV-19 (Covishield Serum Institute India) vaccines to have a higher risk of major AEs [OR 4.2 (1.7–10.4), *p* = 0.002 and OR 33.7 (3.0–374.3), *p* = 0.004] and hospitalizations [OR 4.6 (1.2–18.3), *p* = 0.030 and OR 5.9 (1.5–23.2), *p* = 0.011] compared to other vaccines, though reassuringly, hospitalizations were rare (*n* = 5 and *n* = 4, respectively). These results should be interpreted with caution given the small number of recipients of these vaccines with IIMs (*n* = 15, and *n* = 29, respectively) and wide confidence intervals observed in BLR (Table 4, Suppl. Table 4b, 6b and 4c, 6c).

### Post-COVID-19 vaccination-associated AEs in patients with IIMs, with IBM excluded

Given the relatively lower incidence of AEs among patients with IBM compared to other IIMs subgroups which could skew the risk estimates for AEs in favor of IIMs, we conducted additional analysis excluding these patients, to better understand the risk profile of other IIMs subgroups. It was reassuring to see that patients with IIMs were still less likely to experience joint pain and headache [OR 0.7 (0.5–0.9), *p* = 0.004 and *p* = 0.007, respectively] compared to SAIDs, as well as visual disturbances [OR 0.5 (0.3–0.9), *p* = 0.018], though IIMs subgroups excluding IBM were more likely to develop rashes [OR 1.8 (1.2–2.6), *p* = 0.004] (Suppl. Table 9a, 9c).

Similar characteristics in terms of the risk profile between different vaccines were observed among patients with IIMs excluding IBM. The BNT162b2 (Pfizer) vaccine was associated with a lower risk of rashes [OR 0.5 (0.3–0.8), *p* = 0.009] and major AEs [OR 0.5 (0.3–0.7), *p* = 0.001] (Suppl. Table 9b, 9c). The ChadOx1 nCOV-19 (Oxford/ AstraZeneca) was associated with a more frequent incidence of injection site pain [OR 1.9 (1.1–3.3), *p* = 0.027] and body ache [OR 1.9 (1.0–3.3), *p* = 0.035], as well as other minor AEs, while a higher risk of major AEs [OR 2.7 (1.1–6.8), *p* = 0.037] was observed among recipients of the Sinovac-CoronaVac vaccine (Suppl. Table 9b, 9c).

### Post-COVID-19 vaccination-associated AEs in patients with active and inactive IIM

While the absolute risk of nearly all long-term AEs was higher in patients with an active course of IIM compared to patients with inactive disease, statistically significant differences were only observed in the occurrence of rashes, myalgia, headache, and fatigue (Supplemental table 7). The higher risk of rashes in patients with active IIMs was particularly pronounced [OR 4.7 (1.1–19.7), *p* = 0.033], with the most common being Gottron's signs (*n* = 26) and V signs (*n* = 24). Notably, merely two (0.9%) individuals with inactive IIM developed a rash after vaccination.

### Post-COVID-19 vaccination-associated AEs in patients with only IIM, IIM and non-SAID comorbidity, and IIM with SAID comorbidity

Patients with IIMs and non-SAIDs comorbidities had a comparable risk of any minor and major AEs, and hospitalizations to those with IIM alone, though with a higher risk of joint pain [OR 3.3 (1.5–7.0), *p* = 0.002] and nausea/vomiting [OR 16.8 (1.9–150.8), *p* = 0.012] among patients with non-SAID comorbidities (Suppl. Table 8a and 8b).

In contrast, autoimmune comorbidities conferred a significantly higher risk of delayed-onset AEs among IIMs, with patients with IIMs and co-existing SAIDs being at a fivefold higher risk of experiencing any minor AEs [ OR 5.2 (3.3–8.2), *p* < 0.001], and twice as likely to develop any major AEs [OR 2.1 (1.2–3.8), *p* = 0.008] compared to patients with IIMs alone (Suppl. Table 8a and 8b).

### Post-COVID-19 vaccination-associated AEs in patients with IIM considering the immunosuppressive therapy received

A considerable number of respondents with IIMs and other SAIDs were receiving methotrexate (22.1%), iv or sq IG (14.1%), and rituximab (10.8%) prior to vaccination. Patients on methotrexate therapy were more susceptible to post-vaccination anaphylaxis [OR 3.1 (1.3–7.7) *p* = 0.014], while patients receiving rituximab were more likely to experience difficulty in breathing [OR 2.4 (1.1–5.7), *p* = 0.038], though the absolute numbers of these AEs were small (*n* = 10 and n-8, respectively) (Suppl. Table 5).

### COVID-19 vaccination-associated AEs with a onset of 30 or more days post-vaccination

Minor AEs appearing 30 or more days post-COVID-19 vaccination predominantly included fatigue (64.2%) and myalgia (50.5%), while marked difficulty in breathing (15.8%) was the most common major AE. Among patients with AEs appearing 30 or more days post-vaccination, those with IIMs were less likely to develop joint pain [OR 0.4 (0.2–0.7)] compared to SAIDs (Suppl. Table 10a, 10b), though it was concerning to note that IIMs were more than twice as likely to develop shortness of breath and rash [OR 2.5 (1.3–4.9), *p* = 0.007 and OR 2.7 (1.4–5.2), *p* = 0.002, respectively] (Suppl. Table 10b).

### Characteristics of patients with IIMs requiring hospitalization post-COVID-19 vaccination

Ten patients with IIMs [aged median (IQR) 54.5 (51.25–63.25) years, 7/10 females, 8/10 Caucasians] reported hospitalization potentially related to COVID-19 vaccination, with severe weakness/fatigue (*n* = 4) and dyspnea (*n* = 2) as the most frequent reasons for hospitalization, though most cases appeared to be related to underlying myositis and not a consequence of vaccination. Characteristics of myositis and SAIDs, and HCs requiring hospitalization are detailed in Suppl. Table 12 (12a and 12b).

## Discussion

While the COVID-19 gradually transitions from an acute cause of unprecedented morbidity and mortality to a largely endemic disease in many regions of the world, in a large part due to widespread vaccination efforts, vaccine hesitancy continues to be a significant impediment to achieving optimum vaccination coverage and herd immunity in patients with IIMs, a high-risk group for severe COVID-19 outcomes [20]. Fear of long-term vaccine ADEs may be a cause of this hesitancy, precipitated by a lack of long-term vaccine safety and tolerability data in this patient group from large prospective studies [21].

We reassuringly found a low overall absolute risk of most minor and major vaccine ADEs in patients with IIMs, not exceeding 5% and 3% in most cases, respectively, and hospitalizations were rare. However, the percentage is higher in comparison to the incidence of short-term ADEs explored in a previous analysis from the COVAD study [9]. Notably, patients with IIMs had a lower risk of minor ADEs than other SAIDs, and for certain ADEs, had a lower risk even compared to HCs, but were more prone to develop rashes compared to HCs. Among patients with IIMs, those with active disease, overlap myositis, and receiving ChadOx1 nCOV-19 (Oxford/AstraZeneca) were more vulnerable to ADEs, while those with inclusion body myositis, and BNT162b2 (Pfizer) vaccine recipients were at a relatively lower risk. Autoimmune multimorbidity conferred a higher risk of post-vaccination ADEs in patients with IIMs.

Since certain vaccine ADEs may mimic constitutional symptoms of IIMs, patients with IIM may have found it difficult to differentiate vaccine ADEs from features of their underlying disease, leading to a possible under-reporting of vaccine ADEs such as injection site pain/soreness and fever, explaining the lower risk compared to HCs. Furthermore, the duration of minor ADEs did not differ between patients with IIM and SAIDs. However, if individuals with IIM developed major ADEs, their duration tends to be almost two times longer than in the SAIDs group and almost three times longer than among HCs. This emphasizes the need for close long-term follow-up and monitoring of IIMs patients after COVID-19 vaccination to minimize the delay in required medical care. Particular caution, and perhaps relative contraindication may be warranted in patients with a past history of cardiac and respiratory conditions in anticipation of a possible risk of hospitalization which may be vaccine related.

The higher risk of ADEs in patients with overlap myositis may be explained by the existent burden of not one but several autoimmune disorders with different pathogenesis. However, vaccine safety data in overlap myositis are rather scarce, and this heterogenous group warrants exploration in greater depth. The favorable risk profile of post-vaccine ADEs in IBM patients is consistent with previous studies exploring short-term ADEs [9]. This highlights the heterogeneity in IIMs with a predominance of different pathogenetic patterns across various subtypes [22]. The interferon (IFN) pathway plays a crucial role in myositis-related autoimmune mechanisms [23]. Along with that m,RNA and adenovirus-based vaccines are prone to activate endosomal and cytosolic pattern-recognition receptors (PRRs) [24] and trigger consequently activation of type I interferon production [25]. However, as type I IFN is a key player for the DM subtype, the IBM phenotype depends predominantly on type II IFN involvement [26]. Therefore, it could be a possible explanation for the special status of this IIM subtype.

The association between immunosuppressive treatment and delayed-onset ADEs that was determined in this study should be interpreted with caution, since the numbers were limited. Moreover, certain drugs, such as rituximab, can be prescribed to patients with a more pronounced course or a certain subtype of IIMs.

The most preferred vaccine for patients with IIMs appeared to be BNT162b2 (Pfizer), consistent with recent ACR guidelines [27]. Although recommendations do not suggest one mRNA vaccine over another, our study depicts greater expediency of BNT162b2 (Pfizer) in comparison to mRNA-1273 (Moderna).

Our study explored delayed-onset COVID-19 vaccine adverse events in a large geographically and ethnically diverse sample of patients with a wide range of SAIDs, including large numbers of rare rheumatic diseases, as well as healthy controls, which gives generalizability and reliability to our study. We had a high rate of questionnaire completion and coupled with the patient self-reported anonymized nature of the survey, this offers a unique reflection of the unbiased patient voice.

However, owing to the patient self-reported design, our study had the limitations of recall and reporting bias, convenience sampling, and the plausible underrepresentation of low-income patients without internet access and the severely disabled. Additionally, individuals of African American or African origin and Native American/Indigenous/Pacific Islander ethnicity are under-represented in the cohort.

Nevertheless, our study provides valuable insights into long-term ADEs of COVID-19 vaccination in the vulnerable patient group of IIMs, which is understudied in the current literature, and supports that the benefits of vaccination in reducing severe COVID-19 outcomes in these patients outweigh the risk of potential AEs.

## Conclusion

Vaccination appeared to be reassuringly safe in patients with IIMs in the long term, with most delayed-onset AEs minor, comparable to other SAIDs, and limited to those with co-existent autoimmune diseases and active disease. These observations may be useful in informing guidelines to identify subgroups that warrant close monitoring post-vaccination in anticipation of AEs, while mitigating hesitancy and improving vaccination rates.

## Supplementary Information

Below is the link to the electronic supplementary material.

Supplementary file1 (DOCX 166 KB)

## Data Availability

The datasets generated and/or analyzed during the current study are not publicly available but are available from the corresponding author upon reasonable request.

## Declarations

### Disclaimer

No part of this manuscript has been copied or published elsewhere either in whole or in part.
