# Supplementary material for: Long-term safety of COVID vaccination in individuals with idiopathic inflammatory myopathies: results from the COVAD study
Source: Rheumatol Int. 2023 Jun 23;43(9):1651–64. doi: 10.1007/s00296-023-05345-y (PMC10348925; doi:10.1007/s00296-023-05345-y)
Supplement: Supplementary file 1 — Supplementary file1 (DOCX 166 KB) [file 296_2023_5345_MOESM1_ESM.docx]

SUPPLEMENT

**Supplementary Table 1. Continuation of Table 1 (Baseline characteristics)**

| **Variable** | **Total** | | **IIM** | | **SAIDs** | | **HC** | |
| --- | --- | --- | --- | --- | --- | --- | --- | --- |
|  | **N (8759)** | **% (100)** | **N (1390)** | **% (100)** | **N (4432)** | **% (100)** | **N (2937)** | **% (100)** |
| **SAIDs** | | | | | | | | |
| Ankylosing spondylitis | 282 | 3.2 | 13 | 0.9 | 269 | 6.1 | **-** |  |
| Eosinophilic fasciitis | 6 | 0.1 | 1 | 0.1 | 5 | 0.1 | **-** |  |
| Hemolytic anemia/ ITP | 61 | 0.7 | 15 | 1.1 | 46 | 1.0 | **-** |  |
| IBD | 127 | 1.4 | 0 | 0.0 | 127 | 2.9 | **-** |  |
| MCTD | 121 | 1.4 | 47 | 3.4 | 74 | 1.7 | **-** |  |
| Morphea | 21 | 0.2 | 4 | 0.3 | 17 | 0.4 | **-** |  |
| Multiple sclerosis | 35 | 0.4 | 6 | 0.4 | 29 | 0.7 | **-** |  |
| Myasthenia gravis | 32 | 0.4 | 8 | 0.6 | 24 | 0.5 | **-** |  |
| Myositis or ASS | 925 | 10.6 | 925 | 66.5 | 0 | 0.0 | **-** |  |
| Other AIRDs | 1266 | 14.5 | 465 | 33.5 | 801 | 18.1 | **-** |  |
| Overlap myositis | 214 | 2.4 | 214 | 15.4 | 0 | 0.0 | **-** |  |
| Pernicious.anemia | 37 | 0.4 | 7 | 0.5 | 30 | 0.7 | **-** |  |
| PMR | 146 | 1.7 | 12 | 0.9 | 134 | 3.0 | **-** |  |
| PsA | 241 | 2.8 | 8 | 0.6 | 233 | 5.3 | **-** |  |
| RA | 1646 | 18.8 | 92 | 6.6 | 1554 | 35.1 | **-** |  |
| Scleroderma | 423 | 4.8 | 93 | 6.7 | 330 | 7.4 | **-** |  |
| Sjogren’s | 1101 | 12.6 | 57 | 4.1 | 1044 | 23.6 | **-** |  |
| SLE | 319 | 3.6 | 44 | 3.2 | 275 | 6.2 | **-** |  |
| Thyroid | 787 | 9.0 | 176 | 12.7 | 611 | 13.8 | **-** |  |
| Type1 Diabetes | 78 | 0.9 | 14 | 1.0 | 64 | 1.4 | **-** |  |
| UCTD | 95 | 1.1 | 14 | 1.0 | 81 | 1.8 | **-** |  |
| Vasculitis | 246 | 2.8 | 30 | 2.2 | 216 | 4.9 | **-** |  |
| **Other (non-autoimmune) Comorbidity** | | | | | | | | |
| Asthma | 874 | 10.0 | 205 | 14.7 | 468 | 10.6 | 201 | 6.8 |
| Chronic Kidney Disease | 278 | 3.2 | 50 | 3.6 | 208 | 4.7 | 20 | 0.7 |
| Chronic Liver Disease | 85 | 1.0 | 22 | 1.6 | 53 | 1.2 | 10 | 0.3 |
| Chronic Obstructive Pulmonary Disease (COPD) (Emphysema, Bronchitis, etc.) | 182 | 2.1 | 55 | 4.0 | 103 | 2.3 | 24 | 0.8 |
| Interstitial Lung Disease (ILD) | 387 | 4.4 | 243 | 17.5 | 137 | 3.1 | 7 | 0.2 |
| Coronary Heart Disease | 236 | 2.7 | 110 | 7.9 | 97 | 2.2 | 29 | 1.0 |
| Diabetes | 583 | 6.7 | 190 | 13.7 | 277 | 6.3 | 116 | 3.9 |
| Epilepsy | 69 | 0.8 | 7 | 0.5 | 54 | 1.2 | 8 | 0.3 |
| Hyperlipidemia | 1093 | 12.5 | 354 | 25.5 | 540 | 12.2 | 199 | 6.8 |
| HIV-AIDS | 23 | 0.3 | 7 | 0.5 | 9 | 0.2 | 7 | 0.2 |
| Hypertension | 1560 | 17.8 | 459 | 33.0 | 823 | 18.6 | 278 | 9.5 |
| Stroke | 70 | 0.8 | 28 | 2.0 | 35 | 0.8 | 7 | 0.2 |
| Tuberculosis | 63 | 0.7 | 7 | 0.5 | 45 | 1.0 | 11 | 0.4 |
| Organ Transplant | 26 | 0.3 | 2 | 0.1 | 19 | 0.4 | 5 | 0.2 |
| Other | 407 | 4.6 |  |  |  |  |  |  |

**Supplementary Table 2. Differences in immunosuppressive treatment in IIM and SAID groups**

|  | **IIM** | | **SAIDs** | | **OR (95% CI)** | **p** |
| --- | --- | --- | --- | --- | --- | --- |
|  | **N (1390)** | **% (100)** | **N (4432)** | **% (100)** |  |  |
| Methotrexate | 306 | 22.1 | 1162 | 26.2 | 0.8 (0.7-0.9) | 0.002 |
| Mycophenolate mofetil or mycophenolic acid | 259 | 18.6 | 359 | 8.1 | 2.6 (2.1-3.1) | <0.001 |
| Azathioprine | 133 | 9.6 | 321 | 7.2 | 1.4 (1.1-1.7) | 0.005 |
| Hydroxychloroquine | 226 | 16.3 | 1301 | 29.4 | 0.5 (0.4-0.5) | <0.001 |
| Sulfasalazine | 17 | 1.2 | 306 | 6.9 | 0.2 (0.1-0.3) | <0.001 |
| Leflunomide | 12 | 0.9 | 221 | 5.0 | 0.2 (0.1-0.3) | <0.001 |
| Oral Tacrolimus | 25 | 1.8 | 36 | 0.8 | 2.2 (1.3-3.7) | 0.002 |
| Cyclosporine | 32 | 2.3 | 61 | 1.4 | 1.7 (1.1-2.6) | 0.016 |
| IV immunoglobulin (IVIg) or subcutaneous immunoglobulin (SQIg) | 196 | 14.1 | 22 | 0.5 | 32.9 (21.1-51.3) | <0.001 |
| Cyclophosphamide (Cytoxan) | 13 | 0.9 | 50 | 1.1 |  | NS |
| Rituximab | 149 | 10.8 | 182 | 4.1 | 2.8 (2.3-3.5) | <0.001 |
| Anti TNF agents (infliximab, adalimumab, certolizumab, golimumab, etanercept) | 15 | 1.1 | 356 | 8.0 | 0.1 (0.07-0.21) | <0.001 |
| No steroids | 805 | 57.9 | 2924 | 66.0 | 0.7 (0.6-0.8) | <0.001 |
| <10 mg/day steroids | 366 | 26.3 | 1107 | 25.0 |  | NS |
| 10-20 mg/day steroids | 118 | 8.5 | 276 | 6.2 | 1.4 (1.1-1.7) | 0.003 |
| >20 mg/day steroids | 89 | 6.4 | 94 | 2.1 | 3.1 (2.3-4.2) | <0.001 |

**Supplementary Table** **3.** **Vaccination overview**

|  | Number of doses received | | | | | | | |  | |
| --- | --- | --- | --- | --- | --- | --- | --- | --- | --- | --- |
|  | 1 dose | % | 2 doses | % | 3 doses | % | 4 doses | % |  |  |
| Number of respondents completed the survey | 8759 | 100.0 | 8489 | 96.9 | 6311 | 72.1 | 1381 | 15.8 |  |  |
|  | Number of vaccine doses received by respondents | | | | | | | | | |
|  | With 1 dose | % | With 2 doses | % | With 3 doses | % | With 4 doses | % | Total | % |
| **Pfizer-BioNTech** | 53 | 19.6 | 1251 | 28.7 | 7508 | 50.8 | 2899 | 52.5 | 11711 | **47.0** |
| **Oxford/Astra Zeneca** | 38 | 14.1 | 664 | 15.2 | 2856 | 19.3 | 1038 | 18.8 | 4596 | **18.4** |
| Johnson & Johnson | 89 | 33.0 | 107 | 2.5 | 84 | 0.6 | 26 | 0.5 | 306 | 1.2 |
| **Moderna** | 17 | 6.3 | 438 | 10.1 | 2173 | 14.7 | 838 | 15.2 | 3466 | **13.9** |
| Novavax | 3 | 1.1 | 6 | 0.1 | 6 | 0.0 | 13 | 0.2 | 28 | 0.1 |
| Covishield (Serum Institute of India) | 17 | 6.3 | 729 | 16.7 | 267 | 1.8 | 12 | 0.2 | 1025 | 4.1 |
| Covaxin (Bharat Biotech) | 1 | 0.4 | 113 | 2.6 | 43 | 0.3 | 4 | 0.1 | 161 | 0.7 |
| Sputnik | 3 | 1.1 | 150 | 3.4 | 375 | 2.5 | 52 | 0.9 | 580 | 2.3 |
| Sinopharm | 17 | 6.3 | 454 | 10.4 | 565 | 3.8 | 121 | 2.2 | 1157 | 4.6 |
| Sinovac-CoronaVac | 16 | 5.9 | 272 | 6.2 | 577 | 3.9 | 454 | 8.2 | 1319 | 5.3 |
| I am not sure | 5 | 1.9 | 74 | 1.7 | 55 | 0.4 | 39 | 0.7 | 173 | 0.7 |
| Other | 11 | 4.1 | 98 | 2.3 | 281 | 1.9 | 28 | 0.5 | 418 | 1.7 |

**Supplementary Table 4 Comparison of AEs appearance among IIM, SAID and HC groups considering vaccine was used**

**Supplementary Table** 4 a

| BNT162b2 (Pfizer) | **IIM** | | **SAIDs** | | **HCs** | | **OR1** | **OR2** |
| --- | --- | --- | --- | --- | --- | --- | --- | --- |
|  | N (883) | 100% | N (2939) | 100% | N (1532) | 100% |  |  |
| **Injection site (arm) pain and soreness** | 65 | 7.4 | 371 | 12.6 | 163 | 10.6 | 0.6 (0,4-0,7)*** | 0.7(0.5-0.9)* |
| Myalgia | 59 | 6.7 | 299 | 10.2 | 102 | 6.7 | 0.6 (0.5-0.8)** |  |
| Body ache | 52 | 5.9 | 339 | 11.5 | 116 | 7.6 | 0.5 (0.4-0.7)*** |  |
| Joint pain | 41 | 4.6 | 338 | 11.5 | 90 | 5.9 | 0.5 (0.4-0.7)*** |  |
| Fever | 44 | 5.0 | 240 | 8.2 | 107 | 7.0 | 0.5 (0.4-0.8)*** | 0.6(0.4-0.9)* |
| Chills | 13 | 1.5 | 216 | 7.3 | 76 | 5.0 | 0.7 (0.5-0,9)* |  |
| Cough | 22 | 2.5 | 82 | 2.8 | 25 | 1.6 | 0.5 (0.3-0.9) |  |
| Difficulty in breathing or Shortness of breath | 13 | 1.5 | 81 | 2.8 | 28 | 1.8 |  |  |
| Nausea/vomiting | 55 | 6.2 | 113 | 3.8 | 19 | 1.2 | 0.4 (0.2-0.7)** |  |
| Headache | 24 | 2.7 | 306 | 10.4 | 109 | 7.1 | 0.6 (0.4-0.8)*** |  |
| Rash | 71 | 8.0 | 81 | 2.8 | 16 | 1.0 |  | 2.6 (1.4-5.0)** |
| Fatigue | 16 | 1.8 | 364 | 12.4 | 95 | 6.2 | 0.6 (0.5-0.8)*** |  |
| Diarrhoea | 10 | 1.1 | 82 | 2.8 | 19 | 1.2 |  |  |
| Abdominal pain | 25 | 2.8 | 65 | 2.2 | 19 | 1.2 | 0.5 (0.3-1.0)* |  |
| High pulse rate or palpitations | 13 | 1.5 | 113 | 3.8 | 33 | 2.2 |  |  |
| Rise in blood pressure | 2 | 0.2 | 57 | 1.9 | 16 | 1.0 |  |  |
| Fainting | 29 | 3.3 | 14 | 0.5 | 7 | 0.5 |  |  |
| Dizziness | 10 | 1.1 | 150 | 5.1 | 34 | 2.2 | 0.6 (0.4-0.9)* |  |
| Chest pain | 13 | 1.5 | 75 | 2.6 | 16 | 1.0 |  |  |
| Swelling in the extremities | 27 | 3.1 | 62 | 2.1 | 21 | 1.4 | 0.4 (0.2-0.9)* |  |
| Weakness and tingling in the feet and legs | 21 | 2.4 | 104 | 3.5 | 38 | 2.5 |  |  |
| Pricking or pins and needles sensations in the hands and feet | 10 | 1.1 | 94 | 3.2 | 27 | 1.8 |  |  |
| Visual disturbances (loss of vision, blurring of vision, etc.) | 10 | 1.1 | 76 | 2.6 | 15 | 1.0 |  |  |
| Bleeding/bruising on the body | 3 | 0.3 | 41 | 1.4 | 9 | 0.6 |  |  |
| Petechial rash | 72 | 8.2 | 33 | 1.1 | 7 | 0.5 | 0.3 (0.09-1.0)* |  |
| Anaphylaxis | 11 | 1.2 | 43 | 1.5 | 20 | 1.3 |  |  |
| Marked difficulty in breathing | 26 | 2.9 | 85 | 2.9 | 36 | 2.3 |  |  |
| Throat closure | 13 | 1.5 | 41 | 1.4 | 16 | 1.0 |  |  |
| Severe rashes | 19 | 2.2 | 71 | 2.4 | 29 | 1.9 |  |  |
| **Hospitalisation** | 21 | 2.4 | 113 | 3.8 | 33 | 2.2 | 0.6 (0.4-1.0)* |  |

*P < .05, **P < .005, ***P < .001.

OR 1and 2 compares AEs between IIM and SAIDs, and IIM and HCs respectively.

**Supplementary Table** 4 b

| Sinovac-CoronaVac | **IIM** | | **SAIDs** | | **HC** | | **OR1** | **OR2** |
| --- | --- | --- | --- | --- | --- | --- | --- | --- |
|  | N (29) | %  100 | N (361) | %  100 | N (363) | %  100 |  |  |
| **Injection site (arm) pain and soreness** | 6 | 20.7 | 45 | 12.5 | 37 | 10.2 |  |  |
| Myalgia | 5 | 17.2 | 33 | 9.1 | 27 | 7.4 |  |  |
| Body ache | 6 | 20.7 | 43 | 11.9 | 34 | 9.4 |  |  |
| Joint pain | 5 | 17.2 | 36 | 10.0 | 15 | 4.1 |  | 4.8 (1.6-14.3)** |
| Fever | 5 | 17.2 | 40 | 11.1 | 32 | 8.8 |  |  |
| Chills | 3 | 10.3 | 19 | 5.3 | 22 | 6.1 |  |  |
| Cough | 3 | 10.3 | 6 | 1.7 | 4 | 1.1 | 6.8 (1.6-28.9)** | 10.3 (2.2-48.5)*** |
| Difficulty in breathing or Shortness of breath | 2 | 6.9 | 14 | 3.9 | 6 | 1.7 |  |  |
| Nausea/vomiting | 0 | 0 | 19 | 5.3 | 10 | 2.8 |  |  |
| Headache | 3 | 10.3 | 39 | 10.8 | 28 | 7.7 |  |  |
| Rash | 2 | 6.9 | 13 | 3.6 | 1 | 0.3 |  | 26.7 (2.3-303.5)*** |
| Fatigue | 4 | 13.8 | 41 | 11.4 | 22 | 6.1 |  |  |
| Diarrhoea | 1 | 3.4 | 9 | 2.5 | 2 | 0.6 |  |  |
| Abdominal pain | 3 | 10.3 | 12 | 3.3 | 4 | 1.1 |  | 10.3 (2.1-48.5)*** |
| High pulse rate or palpitations | 4 | 13.8 | 18 | 5.0 | 10 |  | 3.0 (1.0-9.7)* | 5.6 (1.6-19.2)** |
| Rise in blood pressure | 1 | 3.4 | 6 | 1.7 | 3 | 0.8 |  |  |
| Fainting | 2 | 6.9 | 3 | 0.8 | 1 | 0.3 | 8.8 (1.4-55.2)** | 26.7 (2.3-303.5)*** |
| Dizziness | 4 | 13.8 | 22 | 6.1 | 13 | 3.6 |  | 4.3 (1.3-14.1)* |
| Chest pain | 1 | 3.4 | 14 | 3.9 | 8 | 2.2 |  |  |
| Swelling in the extremities | 2 | 6.9 | 10 | 2.8 | 4 | 1.1 |  | 6.6 (1.2-37.7)* |
| Weakness and tingling in the feet and legs | 4 | 13.8 | 16 | 4.4 | 5 | 1.4 | 3.5 (1.1-11.1)* | 11.4 (2.8-45.1)*** |
| Pricking or pins and needles sensations in the hands and feet | 5 | 17.2 | 13 | 3.6 | 3 | 0.8 | 5.6 (1.8-16.9)*** | 24.9 (5.6-110.3)*** |
| Visual disturbances (loss of vision, blurring of vision, etc.) | 2 | 6.9 | 6 | 1.7 | 2 | 0.6 |  | 13.3 (1.8-98.1)** |
| Bleeding/bruising on the body | 2 | 6.9 | 6 | 1.7 | 2 | 0.6 |  | 13.3 (1.8-98.1)** |
| Petechial rash | 2 | 6.9 | 4 | 1.1 | 0 | 0.0 | 6.6 (1.2-37.7)* |  |
| Anaphylaxis | 5 | 17.2 | 7 | 1.9 | 6 | 1.7 | 10.5 (3.1-35.7)*** | 12.3 (3.5-43.3)*** |
| Marked difficulty in breathing | 4 | 13.8 | 13 | 3.6 | 12 | 3.3 | 4.3 (1.3-14.1)* | 4.7 (1.4-15.5)* |
| Throat closure | 5 | 17.2 | 12 | 3.3 | 5 | 1.4 | 6.1 (2.0-18.6)*** | 14.8 (4.0-54.8)*** |
| Severe rashes | 6 | 20.7 | 11 | 3.0 | 8 | 2.2 | 8.3 (2.8-24.5)*** | 11.5 (3.7-36.0)*** |
| **Hospitalisation** | 5 | 17.2 | 11 | 3.0 | 4 | 1.1 | 6.6 (2.1-20.6)*** | 18.6 (4.7-73.8)*** |

*P < .05, **P < .005, ***P < .001.

OR 1and 2 compares AEs between IIM and SAIDs, and IIM and HCs respectively.

**Supplementary Table** 4 c

| ChAdOx1 nCoV-19 (Covishield Serum Institute India) | **IIM** | | **SAIDs** | | **HC** | | **OR1** | **OR2** |
| --- | --- | --- | --- | --- | --- | --- | --- | --- |
|  | N (15) | %  100 | N (175) | %  100 | N (281) | %  100 |  |  |
| **Injection site (arm) pain and soreness** | 4 | 26.7 | 19 | 8.9 | 36 | 12.8 |  |  |
| Myalgia | 1 | 6.7 | 15 | 7.0 | 15 | 5.3 |  |  |
| Body ache | 2 | 13.3 | 19 | 8.9 | 17 | 6.0 |  |  |
| Joint pain | 2 | 13.3 | 12 | 5.6 | 9 | 3.2 |  | 4.7 (0.9-23.7)* |
| Fever | 2 | 13.3 | 12 | 5.6 | 27 | 9.6 |  |  |
| Chills | 1 | 6.7 | 6 | 2.8 | 4 | 1.4 |  |  |
| Cough | 1 | 6.7 | 2 | 0.9 | 1 | 0.4 |  | 20.0 (1.2-336.6)* |
| Difficulty in breathing or Shortness of breath | 2 | 13.3 | 2 | 0.9 | 1 | 0.4 | 16.3 (2.1-125.2)*** | 43.1 (3.7-506.2)*** |
| Nausea/vomiting | 0 | 0 | 3 | 1.4 | 3 | 1.1 |  |  |
| Headache | 0 | 0 | 3 | 1.4 | 8 | 2.8 |  |  |
| Rash | 3 | 20 | 4 | 1.9 | 1 | 0.4 | 13.1 (2.6-65.4)*** | 70.0 (6.8-723.6)*** |
| Fatigue | 2 | 13.3 | 8 | 3.7 | 7 | 2.5 |  | 6.0(1.1-31.9)* |
| Diarrhoea | 1 | 6.7 | 1 | 0.5 | 1 | 0.4 | 15.2 (0.9-256.3)* | 20.0 (1.2-336.6)** |
| Abdominal pain | 1 | 6.7 | 2 | 0.9 | 2 | 0.7 |  | 10.0 (0.9-116.6)* |
| High pulse rate or palpitations | 2 | 13.3 | 4 | 1.9 | 2 | 0.7 | 8.1 (1.4-48.3)* | 21.5(2.8-164.6)*** |
| Rise in blood pressure | 0 | 0 | 1 | 0.5 | 1 | 0.4 |  |  |
| Fainting | 1 | 6.7 | 2 | 0.9 | 1 | 0.4 |  | 20.0(1.2-336.6)** |
| Dizziness | 1 | 6.7 | 3 | 1.4 | 1 | 0.4 |  | 20.0(1.2-336.6)** |
| Chest pain | 0 | 0 | 2 | 0.9 | 0 | 0.0 |  |  |
| Swelling in the extremities | 1 | 6.7 | 3 | 1.4 | 1 | 0.4 |  | 20.0(1.2-336.6)** |
| Weakness and tingling in the feet and legs | 1 | 6.7 | 2 | 0.9 | 1 | 0.4 |  | 20.0(1.2-336.6)** |
| Pricking or pins and needles sensations in the hands and feet | 1 | 6.7 | 1 | 0.5 | 1 | 0.4 | 15.2 (0.9-256.3)* | 20.0(1.2-336.6)** |
| Visual disturbances (loss of vision, blurring of vision, etc.) | 1 | 6.7 | 2 | 0.9 | 2 | 0.7 |  | 10.0 (0.9-116.6)* |
| Bleeding/bruising on the body | 1 | 6.7 | 2 | 0.9 | 2 | 0.7 |  | 10.0 (0.9-116.6)* |
| Petechial rash | 1 | 6.7 | 2 | 0.9 | 1 | 0.4 |  | 20.0(1.2-336.6)** |
| Anaphylaxis | 3 | 20 | 0 | 0.0 | 10 | 3.6 |  | 6.8(1.6-27.9)** |
| Marked difficulty in breathing | 5 | 33.3 | 2 | 0.9 | 6 | 2.1 | 53.0(9.1-307.5)*** | 22.9 (6.0-87.9)*** |
| Throat closure | 3 | 20 | 1 | 0.5 | 6 | 2.1 | 53.3 (5.1-551.0)*** | 11.5(2.6-51.4)*** |
| Severe rashes | 4 | 26.7 | 1 | 0.5 | 8 | 2.8 | 77.5 (8.0-752.4)*** | 12.4 (3.2-47.5)*** |
| **Hospitalisation** | 4 | 26.7 | 2 | 0.9 | 8 | 2.8 | 38.5 (6.4-233.7)*** | 12.4 (3.2-47.5)*** |

*P < .05, **P < .005, ***P < .001.

OR 1and 2 compares AEs between IIM and SAIDs, and IIM and HCs respectively.

**Supplementary Table** 4 d

| MRNA-1273 (Moderna) | **IIM** | | **SAIDs** | | **HC** | | **OR1** | **OR2** |
| --- | --- | --- | --- | --- | --- | --- | --- | --- |
|  | **N (555)** | **100%** | **N (883)** | **100%** | **N (442)** | **100%** | **N (15)** | **N (29)** |
| **Injection site (arm) pain and soreness** | 14 | 2.5 | 109 | 12.3 | 88 | 19.9 |  |  |
| Myalgia | 8 | 1.4 | 74 | 8.4 | 32 | 7.2 |  |  |
| Body ache | 8 | 1.4 | 82 | 9.3 | 36 | 8.1 |  |  |
| Joint pain | 8 | 1.4 | 84 | 9.5 | 20 | 4.5 |  |  |
| Fever | 5 | 0.9 | 77 | 8.7 | 29 | 6.6 | 0.6 (0.4-0.9)* |  |
| Chills | 6 | 1.1 | 55 | 6.2 | 26 | 5.9 |  |  |
| Cough | 1 | 0.2 | 21 | 2.4 | 8 | 1.8 |  |  |
| Difficulty in breathing or Shortness of breath | 4 | 0.7 | 18 | 2.0 | 6 | 1.4 |  |  |
| Nausea/vomiting | 1 | 0.2 | 27 | 3.1 | 7 | 1.6 |  |  |
| Headache | 5 | 0.9 | 69 | 7.8 | 23 | 5.2 | 0.6 (0.4-1.0)* |  |
| Rash | 4 | 0.7 | 36 | 4.1 | 5 | 1.1 |  | 4.1 (1.6-10.9)** |
| Fatigue | 6 | 1.1 | 97 | 11.0 | 29 | 6.6 |  |  |
| Diarrhoea | 2 | 0.4 | 26 | 2.9 | 9 | 2.0 |  |  |
| Abdominal pain | 2 | 0.4 | 13 | 1.5 | 2 | 0.5 |  |  |
| High pulse rate or palpitations | 1 | 0.2 | 24 | 2.7 | 12 | 2.7 |  |  |
| Rise in blood pressure | 1 | 0.2 | 20 | 2.3 | 7 | 1.6 |  |  |
| Fainting | 1 | 0.2 | 5 | 0.6 | 0 | 0.0 |  |  |
| Dizziness | 2 | 0.4 | 38 | 4.3 | 10 | 2.3 |  |  |
| Chest pain | 1 | 0.2 | 19 | 2.2 | 5 | 1.1 |  |  |
| Swelling in the extremities | 2 | 0.4 | 17 | 1.9 | 2 | 0.5 |  |  |
| Weakness and tingling in the feet and legs | 2 | 0.4 | 27 | 3.1 | 7 | 1.6 |  |  |
| Pricking or pins and needles sensations in the hands and feet | 1 | 0.2 | 25 | 2.8 | 8 | 1.8 |  |  |
| Visual disturbances (loss of vision, blurring of vision, etc.) | 2 | 0.4 | 23 | 2.6 | 6 | 1.4 |  |  |
| Bleeding/bruising on the body | 1 | 0.2 | 16 | 1.8 | 2 | 0.5 | 0.2(0,05-0.9)* |  |
| Petechial rash | 1 | 0.2 | 13 | 1.5 | 2 | 0.5 |  |  |
| Anaphylaxis | 9 | 1.6 | 11 | 1.2 | 8 | 1.8 |  |  |
| Marked difficulty in breathing | 10 | 1.8 | 20 | 2.3 | 14 | 3.2 |  |  |
| Throat closure | 9 | 1.6 | 12 | 1.4 | 7 | 1.6 | 2.2 (1.0-4.6)* |  |
| Severe rashes | 10 | 1.8 | 25 | 2.8 | 9 | 2.0 |  |  |
| **Hospitalisation** | 11 | 2 | 74 | 8.4 | 31 | 7.0 | 0.5 (0.3-0.7)** | 0.5 (0.3-1.0)* |

*P < .05, **P < .005, ***P < .001.

OR 1and 2 compares AEs between IIM and SAIDs, and IIM and HCs respectively.

**Supplementary Table** 4 e

| ChadOx1 nCOV-19 (Oxford/ AstraZeneca) | **IIM** | | **SAIDs** | | **HC** | | **OR1** | **OR2** |
| --- | --- | --- | --- | --- | --- | --- | --- | --- |
|  | **N (175)** | **100%** | **N (1507)** | **100%** | **N (897)** | **100%** |  |  |
| **Injection site (arm) pain and soreness** | 38 | 21.7 | 226 | 15.0 | 105 | 11.7 |  |  |
| Myalgia | 18 | 10.3 | 174 | 11.5 | 74 | 8.2 |  |  |
| Body ache | 20 | 11.4 | 208 | 13.8 | 83 | 9.3 |  |  |
| Joint pain | 17 | 9.7 | 195 | 12.9 | 63 | 7.0 |  |  |
| Fever | 15 | 8.6 | 155 | 10.3 | 86 | 9.6 |  |  |
| Chills | 13 | 7.4 | 129 | 8.6 | 72 | 8.0 |  |  |
| Cough | 1 | 0.6 | 50 | 3.3 | 22 | 2.5 | 0.2 (0.02-1.2)* |  |
| Difficulty in breathing or Shortness of breath | 4 | 2.3 | 49 | 3.3 | 19 | 2.1 |  |  |
| Nausea/vomiting | 5 | 2.9 | 71 | 4.7 | 19 | 2.1 |  |  |
| Headache | 17 | 9.7 | 182 | 12.1 | 73 | 8.1 |  |  |
| Rash | 3 | 1.7 | 54 | 3.6 | 11 | 1.2 |  |  |
| Fatigue | 21 | 12 | 204 | 13.5 | 70 | 7.8 |  |  |
| Diarrhoea | 6 | 3.4 | 49 | 3.3 | 12 | 1.3 |  | 2.6 (1.0-7.1)* |
| Abdominal pain | 4 | 2.3 | 29 | 1.9 | 8 | 0.9 |  |  |
| High pulse rate or palpitations | 5 | 2.9 | 64 | 4.2 | 23 | 2.6 |  |  |
| Rise in blood pressure | 6 | 3.4 | 25 | 1.7 | 10 | 1.1 |  | 3.1 (1.1-8.8)* |
| Fainting | 2 | 1.1 | 11 | 0.7 | 5 | 0.6 |  |  |
| Dizziness | 8 | 4.6 | 97 | 6.4 | 18 | 2.0 |  | 2.3 (1.0-5.5)* |
| Chest pain | 3 | 1.7 | 47 | 3.1 | 14 | 1.6 |  |  |
| Swelling in the extremities | 6 | 3.4 | 41 | 2.7 | 11 | 1.2 |  | 2.9 (1.0-7.8)* |
| Weakness and tingling in the feet and legs | 10 | 5.7 | 59 | 3.9 | 19 | 2.1 |  | 2.8 (1.3-6.1)* |
| Pricking or pins and needles sensations in the hands and feet | 8 | 4.6 | 47 | 3.1 | 15 | 1.7 |  | 2.8 (1.2-6.7)* |
| Visual disturbances (loss of vision, blurring of vision, etc.) | 5 | 2.9 | 47 | 3.1 | 12 | 1.3 |  |  |
| Bleeding/bruising on the body | 6 | 3.4 | 29 | 1.9 | 5 | 0.6 |  | 6.3 (1.9-21.0)** |
| Petechial rash | 2 | 1.1 | 21 | 1.4 | 2 | 0.2 |  |  |
| Anaphylaxis | 5 | 2.9 | 25 | 1.7 | 10 | 1.1 |  |  |
| Marked difficulty in breathing | 7 | 4 | 44 | 2.9 | 22 | 2.5 |  |  |
| Throat closure | 6 | 3.4 | 22 | 1.5 | 14 | 1.6 |  |  |
| Severe rashes | 6 | 3.4 | 28 | 1.9 | 16 | 1.8 |  |  |
| **Hospitalisation** | 8 | 4.6 | 70 | 4.6 | 29 | 3.2 |  |  |

*P < .05, **P < .005, ***P < .001.

OR 1and 2 compares AEs between IIM and SAIDs, and IIM and HCs respectively.

**Supplementary Table 5. Factors significant in multivariable analysis (BLR) between IIMs, SAIDs, and HCs**

| *IIMs as compared to HCs (adjusted for age, gender, ethnicity, comorbidity, number of doses received, and stratified for country of origin )** | | | | |
| --- | --- | --- | --- | --- |
|  | B coefficient | S.E. | Exp (B) CI (95%) | P value |
| Rash | 1.374 | 0.295 | **4.0 (2.2-7.0)** | **<0.001** |
| Severe rashes | 0.732 | 0.265 | **2.1 (1.2-3.5)** | **0.006** |
| *IIMs as compared to other SAIDs (adjusted for age, gender, ethnicity, comorbidity, number of vaccine doses received, IS drugs, and stratified for country of origin)* | | | | |
| Injection site (arm) pain and soreness | -0.264 | 0.122 | **0.8 (0.6-1.0)** | **0.030** |
| Joints pain | -0.481 | 0.133 | **0.6 (0.5-0.8)** | **<0.001** |
| Headache | -0.432 | 0.138 | **0.6 (0.5-0.9)** | **0.002** |
| Fatigue | -0.293 | 0.119 | **0.7 (0.6-0.9)** | **0.014** |
| Dizziness | -0.425 | 0.188 | **0.7 (0.5-0.9)** | **0.024** |
| *OM vs Rest of IIM (adjusted for age, gender, ethnicity and stratified for country of origin, Vaccine received and IS drugs)* | | | | |
| Minor symptoms | 1.480 | 0.232 | **4.4 (2.8-6.9)** | **<0.001** |
| Injection site (arm) pain and soreness | 0.711 | 0.333 | **2.0 (1.1-3.9)** | **0.033** |
| Myalgia | 1.179 | 0.330 | **3.3 (1.7-6.2)** | **<0.001** |
| Body ache | 1.567 | 0.307 | **4.8 (2.6-8.7)** | **<0.001** |
| Joint pain | 1.556 | 0.339 | **4.7 (2.4-9.2)** | **<0.001** |
| Fever | 1.281 | 0.377 | **3.6 (1.7-7.5)** | **0.001** |
| Chills | 1.264 | 0.377 | **3.5 (1.7-7.4)** | **0.001** |
| Cough | 2.034 | 0.653 | **7.6 (2.1-27.5)** | **0.002** |
| Difficulty | 1.424 | 0.498 | **4.2 (1.6-11.0)** | **0.004** |
| Nausea | 1.462 | 0.552 | **4.3 (1.5-12.7)** | **0.008** |
| Headache | 1.364 | 0.345 | **3.9 (2.0-7.7)** | **<0.001** |
| Rash | 1.498 | 0.425 | **4.5 (1.9-10.3)** | **<0.001** |
| Fatigue | 1.278 | 0.302 | **3.6 (2.0-6.5)** | **<0.001** |
| Diarrhoea | 1.620 | 0.600 | **5.1 (1.6-16.4)** | **0.007** |
| Abdominal pain | 1.596 | 0.652 | **4.9 (1.4-17.7)** | **0.014** |
| High pulse rate or palpitations | 1.777 | 0.486 | **5.9 (2.3-15.3** | **<0.001** |
| Dizziness | 1.368 | 0.486 | **3.9 (1.5-10.2)** | **0.005** |
| Weakness and tingling in the feet and legs | 1.352 | 0.490 | **3.9 (1.5-10.1)** | **0.006** |
| Pricking or pins and needles sensations in the hands and feet | 1.596 | 0.579 | **4.9 (1.6-15.3)** | **0.006** |
| Petechial rash | 2.735 | 0.988 | **15.4 (2.2-106.8)** | **0.006** |
| Major symptoms | 1.418 | 0.278 | **4.1 (2.4-7.1)** | **<0.001** |
| Severe rash | 1.248 | 0.517 | **3.5 (1.3-9.6)** | **0.016** |
| Hospitalization | 1.350 | 0.533 | **3.9 (1.4-11.0)** | **0.011** |
| *IBM vs Rest of IIM (adjusted for age, gender, ethnicity and stratified for country of origin, Vaccine received and IS drugs)* | | | | |
| Minor symptoms | -1.246 | 0.263 | **0.3 (0.2-0.5)** | **<0.001** |
| Myalgia | -1.181 | 0.559 | **0.3 (0.1-0.9)** | **0.035** |
| Joints pain | -1.702 | 0.758 | **0.2 (0.04-0.8)** | **0.025** |
| Rash | -2.417 | 1.051 | **0.1 (0.01-0.7)** | **0.021** |
| Major symptoms | -0.746 | 0.313 | **0.5 (0.3-0.9)** | **0.017** |
| *Post COVID-19 Vaccination associated ADEs in patients with IIM as per IS therapy received (Methotrexate) (adjusted for age, gender, ethnicity, number of vaccine doses received and stratified for country of origin)* | | | | |
| Anaphylaxis | 1.137 | 0.462 | **3.1 (1.3-7.7)** | **0.014** |
| *Post COVID-19 Vaccination associated ADEs in patients with IIM as per IS therapy received (Rituximab) (adjusted for age, gender, ethnicity, number of vaccine doses received and stratified for country of origin)* | | | | |
| Difficulty in breathing, shortness of breath | 0.892 | 0.430 | **2.4 (1.1-5.7)** | **0.038** |

BLR Binary logistic regression, AE Adverse event, SAID systemic autoimmune and inflammatory disorders, DM Dermatomyositis, PM Polymyositis, OM Overlap myositis, IIM Idiopathic inflammatory myopathies, IS – immunosuppressive; HC Healthy control, P<0.05 significant

* After adjustment for IS drugs prescription no statistically significant differences between IIMs and HCs were observed.

**Supplementary Table 6. Overview of BLR results based on vaccine received.**

**Supplementary Table** 6 a

| ***Among BNT162b2 (Pfizer) vaccine recipients***  *IIMs as compared to HCs (adjusted for age, gender, ethnicity, and stratified for country of origin)* | | | | |
| --- | --- | --- | --- | --- |
|  | B coefficient | S.E. | Exp (B) CI (95%) | P value |
| Rash | 1.103 | 0.379 | **3.0 (1.4-6.3)** | **0.004** |
| *IIMs as compared to SAIDs (adjusted for age, gender, ethnicity, and stratified for country of origin)* | | | | |
| Body ache | -0.365 | 0.155 | **0.7 (0.5-0.9)** | **0.019** |
| Joints pain | -0.532 | 0.161 | **0.6 (0.4-0.8)** | **0.001** |
| Nausea/vomiting | -0.669 | 0.305 | **0.5 (0.3-0.9)** | **0.028** |
| Headache | -0.371 | 0.160 | **0.7 (0.5-0.9)** | **0.021** |
| Fatigue | -0.376 | 0.143 | **0.7 (0.5-0.9)** | **0.008** |
| Injection site (arm) pain and soreness | -.0.356 | 0.148 | **0.7 (0.5-0.9)** | **0.016** |
| *IIMs as compared to SAIDs (adjusted for age, gender, ethnicity, number of doses received, dose of IS, and stratified for country of origin)* | | | | |
| Flair of the disease | -.0272 | 0.127 | **0.8 (0.6-1.0)** | **0.032** |
| *OM vs other IIM (adjusted for age, gender, ethnicity, comorbidity, dose of immunosuppressant and stratified by country of origin)* | | | | |
| Body ache | 1.380 | 0.421 | **4.0 (1.7-9.1)** | **0.001** |
| Joint pain | 1.079 | 0.476 | **2.9 (1.2-7.5)** | **0.023** |
| Fever | 1.041 | 0.505 | **2.8 (1.1-7.6)** | **0.039** |
| Chills | 1.195 | 0.476 | **3.3 (1.3-8.4)** | **0.012** |
| Nausea/vomiting | 1.990 | 0.744 | **7.3 (1.7-31.4)** | **0.007** |
| Headache | 1.377 | 0.430 | **4.0 (1.7-9.2)** | **0.001** |
| Rash | 1.751 | 0.611 | **5.8 (1.7-19.1)** | **0.004** |
| Fatigue | 1.170 | 0.401 | **3.2 (1.5-7.1)** | **0.004** |
| High pulse rate or palpitations | 1.476 | 0.581 | **4.4 (1.4-13.7)** | **0.011** |
| Rise in blood pressure | 1.642 | 0.824 | **5.2 (1.0-26.0)** | **0.046** |
| Dizziness | 1.664 | 0.549 | **5.3 (1.8-15.5)** | **0.002** |
| *IBM vs other IIM (adjusted for age, gender, ethnicity, comorbidity, dose of immunosuppressant and stratified by country of origin)* | | | | |
| Throat closure | 2.938 | 1.218 | **18.9 (1.7-205.4)** | **0.016** |
| *BNT162b2 (Pfizer) vs rest of vaccines in IIM patients (adjusted for age, gender, ethnicity, comorbidity, dose of immunosuppressant, number of vaccine doses received and stratified for country of origin)* | | | | |
| Injection site (arm) pain and soreness | -0.437 | 0.212 | **0.6 (0.4-1.0)** | **0.039** |
| Rash | -0.629 | 0.299 | **0.5 (0.3-1.0)** | **0.036** |
| Petechial rash | -1.603 | 0.718 | **0.2 (0.04-0.8)** | **0.026** |
| Major symptoms | -0.472 | 0.193 | **0.6 (0.4-0.9)** | **0.014** |
| Severe diffuse body rash | -0.682 | 0.337 | **0.5 (0.3-1.0)** | **0.043** |

**Supplementary Table** 6b

| ***Among Sinovac-Coronavac vaccine recipients***  *IIMs as compared to HCs (adjusted for age, gender, ethnicity, and stratified for country of origin)* | | | | |
| --- | --- | --- | --- | --- |
|  | B coefficient | S.E. | Exp (B) CI (95%) | P value |
| Joints pain | 1.637 | 0.634 | **5.1 (1.5-17.8)** | **0.010** |
| Cough | 2.862 | 1.013 | **17.5 (2.4-127.4)** | **0.005** |
| Rash | 2.516 | 1.270 | **12.4 (1.0-149.1)** | **0.048** |
| Abdominal pain | 2.465 | 1.054 | **11.8 (1.5-92.9)** | **0.019** |
| High pulse rate or palpitations | 1.926 | 0.726 | **6.9 (1.7-28.5)** | **0.008** |
| Dizziness | 1.360 | 0.674 | **3.9 (1.0-14.6)** | **0.044** |
| Swelling in the extremities | 2.425 | 1.084 | **11.3 (1.4-94.6)** | **0.025** |
| Weakness and tingling in the feet and legs | 2.362 | 0.830 | **10.6 (2.1-54.0)** | **0.004** |
| Pricking or pins and needles sensations in the hands and feet | 3.775 | 1.173 | **43.6 (4.4-433.9)** | **0.001** |
| Bleeding/bruising on the body | 2.795 | 1.365 | **16.4 (1.1-237.7)** | **0.041** |
| Anaphylaxis | 2.693 | 0.845 | **14.8 (2.8-77.4)** | **0.001** |
| Marked difficulty in breathing | 1.549 | 0.757 | **4.7 (1.1-20.7)** | **0.041** |
| Throat closure | 2.380 | 0.757 | **10.8 (2.5-47.6)** | **0.002** |
| Severe rashes | 2.354 | 0.712 | **10.5 (2.6-42.5)** | **0.001** |
| Hospitalization | 3.261 | 0.906 | **26.1 (4.4-154.1)** | **<0.001** |
| *IIMs as compared to SAIDs (adjusted for age, gender, ethnicity, and stratified for country of origin)* | | | | |
| Cough | 1.987 | 0.754 | **7.3 (1.7-32.0)** | **0.008** |
| Fainting | 2.215 | 0.957 | **9.2 (1.4-59.8)** | **0.021** |
| Weakness and tingling in the feet and legs | 1.482 | 0.673 | **4.4 (1.2-16.5)** | **0.028** |
| Pricking or pins and needles sensations in the hands and feet | 1.768 | 0.628 | **5.9 (1.7-20.1)** | **0.005** |
| Anaphylaxis | 2.373 | 0.690 | **10.7 (2.8-41.5)** | **0.001** |
| Marked difficulty in breathing | 1.446 | 0.692 | **4.2 (1.1-16.5)** | **0.037** |
| Throat closure | 1.820 | 0.605 | **6.2 (1.9-20.2)** | **0.003** |
| Severe | 2.278 | 0.623 | **9.8 (2.9-33.1)** | **<0.001** |
| Hospitalization | 1.994 | 0.649 | **7.3 (2.1-26.2)** | **0.002** |
| ***Sinovac-Coronavac*** *vs rest of vaccines in IIM patients (adjusted for age, gender, ethnicity, comorbidity, dose of immunosuppressant, number of vaccine doses received and stratified for country of origin)* | | | | |
| Joints pain | 1.242 | 0.580 | **3.5 (1.1-10.8)** | **0.032** |
| Cough | 2.417 | 0.826 | **11.2 (2.2-56.6)** | **0.003** |
| Abdominal pain | 2.567 | 0.819 | **13.0 (2.6-64.8)** | **0.002** |
| High pulse rate | 1.655 | 0.669 | **5.2 (1.4-19.4)** | **0.013** |
| Fainting | 5.670 | 2.088 | **290.1 (4.8-17377.4)** | **0.007** |
| Dizziness | 1.628 | 0.678 | **5.1 (1.3-19.3)** | **0.016** |
| Weakness and tingling in the feet and legs | 1.886 | 0.705 | **6.6 (1.7-26.2)** | **0.007** |
| Pricking or pins and needles sensations in the hands and feet | 2.721 | 0.734 | **15.2 (3.6-64.1)** | **<0.001** |
| Visual disturbances | 2.871 | 1.020 | **17.7 (2.4-130.4)** | **0.005** |
| Anaphylaxis | 2.158 | 0.776 | **8.7 (1.9-39.6)** | **0.005** |
| Throat closure | 2.084 | 0.750 | **8.0 (1.8-35.0)** | **0.005** |
| Severe rashes | 1.902 | 0.630 | **6.7 (2.0-23.0)** | **0.003** |
| Hospitalization | 1.526 | 0.704 | **4.6 (1.2-18.3)** | **0.030** |
| Major symptoms | 1.430 | 0.463 | **4.2 (1.7-10.4)** | **0.002** |

**Supplementary Table** 6c

| ***Among*** ChAdOx1 nCoV-19 (Covishield Serum Institute India) ***vaccine recipients***  *IIMs as compared to HCs (adjusted for age, gender, ethnicity)* | | | | |
| --- | --- | --- | --- | --- |
|  | B coefficient | S.E. | Exp (B) CI (95%) | P value |
| High pulse rate or palpitations | 3.531 | 1.579 | **34.2 (1.5-754.5)** | **0.025** |
| Hospitalization | 2.877 | 1.096 | **17.8 (2.1-152.0)** | **0.009** |
| Severe rashes | 2.999 | 1.105 | **20.1 (2.3-175.1)** | **0.007** |
| Marked difficulty in breathing | 3.813 | 1.030 | **45.3 (6.0-341.1)** | **<0.001** |
| *IIMs as compared to SAIDs (adjusted for age, gender, ethnicity)* | | | | |
| Flare of AIDs | 2.329 | 0.679 | **10.3 (2.7-38.9)** | **0.001** |
| Rash | 3.143 | 1.128 | **23.2 (2.5-211.1)** | **0.005** |
| Hospitalization | 3.351 | 1.104 | **28.5 (3.3-248.4)** | **0.002** |
| Severe rashes | 4.522 | 1.355 | **92.0 (6.5-1309.2)** | **0.001** |
| Marked difficulty in breathing | 4.278 | 1.072 | **72.1 (8.8-589.4)** | **<0.001** |
| ChAdOx1 nCoV-19 *vs rest of vaccines in IIM patients (adjusted for age, gender, ethnicity)* | | | | |
| Rash | 1.832 | 0.791 | **6.2 (1.3-29.5)** | **0.021** |
| Anaphylaxis | 2.690 | 0.884 | **14.7 (2.6-83.3)** | **0.002** |
| Marked difficulty in breathing | 3.005 | 0.757 | **20.2 (4.6-88.9)** | **<0.001** |
| Throat closure | 2.790 | 0.886 | **16.3 (2.9-92.4)** | **0.002** |
| Severe rashes | 2.159 | 0.724 | **8.7 (2.1-35.8)** | **0.003** |
| Hospitalization | 1.772 | 0.699 | **5.9 (1.5-23.2)** | **0.011** |
| Major symptoms | 3.517 | 1.229 | **33.7 (3.0-374.3)** | **0.004** |

**Supplementary Table** 6 d

| ***Among*** ChadOx1 nCOV-19 (Oxford/ AstraZeneca) ***vaccine recipients***  *IIMs as compared to HCs (adjusted for age, gender, ethnicity, comorbidity, dose, and stratified for country of origin)* | | | | |
| --- | --- | --- | --- | --- |
|  | B coefficient | S.E. | Exp (B) CI (95%) | P value |
| Diarrhoea | 1.385 | 0.612 | **4.0 (1.2-13.3)** | **0.024** |
| Weakness and tingling in the feet and legs | 0.992 | 0.467 | **2.7 (1.1-6.7)** | **0.034** |
| Bleeding/bruising on the body | 1.850 | 0.683 | **6.4 (1.7-24.3)** | **0.007** |
| *OM vs other IIM (adjusted for age, gender, ethnicity, comorbidity, dose of immunosuppressant and stratified by country of origin)* | | | | |
| Chills | 2.957 | 1.231 | **19.2 (1.7-214.9)** | **0.016** |
| Dizziness | 4.549 | 2.217 | **94.6 (1.2-7290.7)** | **0.040** |
| ChadOx1 nCOV-19 (Oxford/ AstraZeneca) *vs rest of vaccines in IIM patients (adjusted for age, gender, ethnicity, comorbidity, dose of immunosuppressant, and stratified for country of origin)* | | | | |
| Headache | 0.786 | 0.307 | **2.2 (1.2-4.0)** | **0.011** |
| Rise in blood pressure | 1.313 | 0.532 | **3.7 (1.3-10.5)** | **0.014** |
| Bleeding/bruising on the body | 1.915 | 0.619 | **6.8 (2.0-22.9)** | **0.002** |

**Supplementary Table** 6 e

| ***Among*** **MRNA-1273 (Moderna)** ***vaccine recipients***  ***OM vs other IIM (adjusted for age, gender, ethnicity, number of vaccine doses received and stratified by country of origin)*** | | | | |
| --- | --- | --- | --- | --- |
|  | B coefficient | S.E. | Exp (B) CI (95%) | P value |
| Injection site (arm) pain and soreness | 1.103 | 0.475 | **3.0 (1.2-7.6)** | **0.020** |
| Myalgia | 1.495 | 0.486 | **4.5 (1.7-11.6)** | **0.002** |
| Body ache | 1.616 | 0.480 | **5.0 (2.0-12.9)** | **0.001** |
| Joint pain | 1.741 | 0.515 | **5.7 (2.1-15.7)** | **0.001** |
| Fever | 1.664 | 0.572 | **5.3 (1.7-16.2)** | **0.004** |
| Cough | 2.310 | 1.058 | **10.1 (1.3-80.2)** | **0.029** |
| Rash | 1.458 | 0.614 | **4.3 (1.3-14.3)** | **0.018** |
| Fatigue | 1.729 | 0.461 | **5.6 (2.3-13.9)** | **<0,001** |
| Diarrhoea | 3.426 | 1.082 | **30.7 (3.7-256.4)** | **0.002** |
| High pulse rate or palpitations | 2.028 | 0.822 | **7.6 (1.5-38.1)** | **0.014** |
| Weakness and tingling in the feet and legs | 1.856 | 0.795 | **6.4 (1.3-30.4)** | **0.020** |
| Visual disturbances | 3.594 | 1.280 | **36.4 (3.0-447.1)** | **0.005** |
| Petechial rash | 3.920 | 1.376 | **50.4 (3.4-747.9)** | **0.004** |
| Severe rashes | 1.621 | 0.664 | **5.1 (1.4-18.6)** | **0.015** |
| MRNA-1273 (Moderna) *vs rest of vaccines in IIM patients (adjusted for age, gender, ethnicity, comorbidity, dose of immunosuppressant, number of vaccine doses received and stratified for country of origin)* | | | | |
| Injection site (arm) pain and soreness | 0.510 | 0.212 | **1.7 (1.1-2.5)** | **0.016** |
| Throat closure | 1.229 | 0.489 | **3.4 (1.3-8.9)** | **0.012** |
| Hospitalization | 1.021 | 0.374 | **2.8 (1.3-5.8)** | **0.006** |
| Severe rashes | 0.774 | 0.346 | **2.2 (1.1-4.3)** | **0.025** |

**Supplementary Table 7. Comparison of vaccination related AEs among active and inactive IIM cases**

|  | **Active IIM** | | **Inactive IIM** | | **Univariable** | | **Multivariable** | |
| --- | --- | --- | --- | --- | --- | --- | --- | --- |
|  | **N 1173** | **%** | **N 217** | **%** | **OR (95% CI)** | **P, value** | **OR (95% CI)** | **P, value** |
| **Minor AEs** | 199 | 17.0 | 28 | 12.9 |  | 0.137 |  |  |
| **Injection site (arm) pain and soreness** | 99 | 8.4 | 16 | 7.4 |  | 0.600 |  |  |
| Myalgia | 94 | 8.0 | 9 | 4.1 | **2.0 (1.0-4.1)** | **0.046** | **2.2 (1.1-4.7)** | **0.036** |
| Body ache | 94 | 8.0 | 14 | 6.5 |  | 0.430 |  |  |
| Joint pain | 80 | 6.8 | 11 | 5.1 |  | 0.338 |  |  |
| Fever | 59 | 5.0 | 12 | 5.5 |  | 0.759 |  |  |
| Chills | 62 | 5.3 | 10 | 4.6 |  | 0.679 |  |  |
| Cough | 19 | 1.6 | 4 | 1.8 |  | 0.813 |  |  |
| Difficulty in breathing or Shortness of breath | 34 | 2.9 | 2 | 0.9 |  | 0.092 |  |  |
| Nausea/vomiting | 26 | 2.2 | 3 | 1.4 |  | 0.430 |  |  |
| Headache | 80 | 6.8 | 6 | 2.8 | **2.6 (1.1-6.0)** | **0.023** | **3.1 (1.2-7.8)** | **0.017** |
| Rash | 53 | 4.5 | 2 | 0.9 | **5.1 (1.2-21.0)** | **0.013** | **4.7 (1.1-19.7)** | **0.033** |
| Fatigue | 113 | 9.6 | 9 | 4.1 | **2.5 (1.2-4.9)** | **0.009** | **2.4 (1.2-4.9)** | **0.015** |
| Diarrhoea | 21 | 1.8 | 4 | 1.8 |  | 0.957 |  |  |
| Abdominal pain | 21 | 1.8 | 3 | 1.4 |  | 0.672 |  |  |
| High pulse rate or palpitations | 31 | 2.6 | 5 | 2.3 |  | 0.773 |  |  |
| Rise in blood pressure | 16 | 1.4 | 3 | 1.4 |  | 0.983 |  |  |
| Fainting | 3 | 0.3 | 1 | 0.5 |  | 0.604 |  |  |
| Dizziness | 37 | 3.2 | 6 | 2.8 |  | 0.761 |  |  |
| Chest pain | 16 | 1.4 | 0 | 0.0 |  | 0.065 |  |  |
| Swelling in the extremities | 18 | 1.5 | 3 | 1.4 |  | 0.580 |  |  |
| Weakness and tingling in the feet and legs | 43 | 3.7 | 4 | 1.8 |  | 0.172 |  |  |
| Pricking or pins and needles sensations in the hands and feet | 34 | 2.9 | 2 | 0.9 |  | 0.092 |  |  |
| Visual disturbances (loss of vision, blurring of vision, etc.) | 17 | 1.4 | 0 | 0.0 |  | 0.055 |  |  |
| Bleeding/bruising on the body | 14 | 1.2 | 0 | 0.0 |  | 0.092 |  |  |
| Petechial rash | 11 | 0.9 | 0 | 0.0 |  | 0.153 |  |  |
| **Major AEs** | 125 | 10.7 | 17 | 7.8 |  | 0.207 |  |  |
| Anaphylaxis | 17 | 1.4 | 3 | 1.4 |  | 0.618 |  |  |
| Marked difficulty in breathing | 42 | 3.6 | 4 | 1.8 |  | 0.189 |  |  |
| Throat closure | 20 | 1.7 | 4 | 1.8 |  | 0.530 |  |  |
| Severe rashes | 35 | 3.0 | 7 | 3.2 |  | 0.848 |  |  |
| **Hospitalisation** | 37 | 3.2 | 4 | 1.8 |  | 0.294 |  |  |

**Supplementary Table 8a. Comparison of vaccination related long-term AEs between respondents with IIM and non-SAID comorbidity, IIM and SAID comorbidity and IIM only.**

|  | **IIM** | | **IIM+ non SAIDs** | | **IIM+ SAIDs** | | **OR1**  **(95%CI)** | **OR2**  **(95%CI)** |
| --- | --- | --- | --- | --- | --- | --- | --- | --- |
|  | N (350) | 100% | N (826) | 100% | N (214) | 100% |  |  |
| **Injection site (arm) pain and soreness** | 17 | 4.9 | 58 | 7.0 | 40 | 18.7 |  | 4.5 (2.5-8.2)***# |
| **Minor symptoms** | 39 | 11.1 | 104 | 12.6 | 84 | 39.3 |  | 5.2 (3.3-7.9)***# |
| Myalgia | 19 | 5.4 | 49 | 5.9 | 35 | 16.4 |  | 3.4 (1.9-6.1)***# |
| Body ache | 17 | 4.9 | 49 | 5.9 | 42 | 19.6 |  | 4.8 (2.6-8.7)***# |
| Joint pain | 10 | 2.9 | 46 | 5.6 | 35 | 16.4 | 2.0 (1.0-4.0)*# | 6.6 (3.2-13.7)***# |
| Fever | 13 | 3.7 | 34 | 4.1 | 24 | 11.2 |  | 3.3 (1.6-6.6)***# |
| Chills | 9 | 2.6 | 39 | 4.7 | 24 | 11.2 |  | 4.8 (2.2-10.5)***# |
| Cough | 4 | 1.1 | 10 | 1.2 | 9 | 4.2 |  | 3.8 (1.2-12.5)*# |
| Difficulty in breathing or Shortness of breath | 3 | 0.9 | 17 | 2.1 | 16 | 7.5 |  | 9.3 (2.7-32.5)***# |
| Nausea/vomiting | 1 | 0.3 | 17 | 2.1 | 11 | 5.1 | 7.3 (1.0-55.3)*# | 18.9 (2.4-147.6)***# |
| Headache | 11 | 3.1 | 44 | 5.3 | 31 | 14.5 |  | 5.2 (2.6-10.6)***# |
| Rash | 12 | 3.4 | 23 | 2.8 | 20 | 9.3 |  | 2.9 (1.4-6.1)**# |
| Fatigue | 16 | 4.6 | 62 | 7.5 | 44 | 20.6 |  | 5.4 (3.0-9.9)***# |
| Diarrhoea | 2 | 0.6 | 15 | 1.8 | 8 | 3.7 |  | 6.8 (1.4-32.1)**# |
| Abdominal pain | 3 | 0.9 | 11 | 1.3 | 10 | 4.7 |  | 5.7 (1.5-20.8)**# |
| High pulse rate or palpitations | 6 | 1.7 | 15 | 1.8 | 15 | 7.0 |  | 4.3 (1.7-11.3)**# |
| Rise in blood pressure | 2 | 0.6 | 10 | 1.2 | 7 | 3.3 |  | 5.9 (1.2-28.6)*# |
| Fainting | 0 | 0.0 | 2 | 0.2 | 2 | 0.9 |  |  |
| Dizziness | 7 | 2.0 | 20 | 2.4 | 16 | 7.5 |  | 4.0 (1.6-9.8)**# |
| Chest pain | 4 | 1.1 | 9 | 1.1 | 3 | 1.4 |  |  |
| Swelling in the extremities | 3 | 0.9 | 15 | 1.8 | 3 | 1.4 |  |  |
| Weakness and tingling in the feet and legs | 8 | 2.3 | 24 | 2.9 | 15 | 7.0 |  | 3.2 (1.3-7.7)*# |
| Pricking or pins and needles sensations in the hands and feet | 4 | 1.1 | 21 | 2.5 | 11 | 5.1 |  | 4.7 (1.5-14.9)**# |
| Visual disturbances (loss of vision, blurring of vision, etc.) | 1 | 0.3 | 11 | 1.3 | 5 | 2.3 |  | 8.3 (1.0-72.0)* |
| Bleeding/bruising on the body | 1 | 0.3 | 11 | 1.3 | 2 | 0.9 |  |  |
| Petechial rash | 0 | 0.0 | 5 | 0.6 | 6 | 2.8 |  |  |
| **Major symptoms** | 31 | 8.9 | 76 | 9.2 | 35 | 16.4 |  | 2.0 (1.2-3.4)*# |
| Anaphylaxis | 3 | 0.9 | 13 | 1.6 | 4 | 1.9 |  |  |
| Marked difficulty in breathing | 7 | 2.0 | 27 | 3.3 | 12 | 5.6 |  | 2.9 (1.1-7.5)*# |
| Throat closure | 2 | 0.6 | 14 | 1.7 | 8 | 3.7 |  | 6.8 (1.4-32.1)*# |
| Severe rashes | 8 | 2.3 | 23 | 2.8 | 11 | 5.1 |  |  |
| **Hospitalisation** | 8 | 2.3 | 22 | 2.7 | 11 | 5.1 |  |  |

#Significant in BLR (binary logistic regression) adjusted for age, gender, ethnicity, and stratified for country of origin. *P < .05, **P < .005, ***P < .001.

**Supplementary Table 8b. Factors significant in multivariable analysis (BLR) between IIM and non-SAID comorbidity, IIM and SAID comorbidity and IIM only.**

| *IIMs with non AIDs as compared to IIMs without comorbidities (adjusted for age, gender, ethnicity, and stratified for country of origin )* | | | | |
| --- | --- | --- | --- | --- |
|  | B coefficient | S.E. | Exp (B) CI (95%) | P value |
| Joint pain | 1.184 | 0.390 | **3.3 (1.5-7.0)** | **0.002** |
| Nausea/vomiting | 2.823 | 1.119 | **16.8 (1.9-150.8)** | **0.012** |
| *IIMs with AIDs as compared to IIMs without comorbidities adjusted for* *age, gender, ethnicity, and stratified for country of origin)* | | | | |
| **Minor symptoms** | 1.647 | 0.233 | **5.2 (3.3-8.2)** | **<0.001** |
| **Injection site (arm) pain and soreness** | 1.554 | 0.320 | **4.7 (2.5-8.9)** | **<0.001** |
| Myalgia | 1.150 | 0.311 | **3.2 (1.7-5.8)** | **<0.001** |
| Body ache | 1.674 | 0.324 | **5.3 (2.8-10.1)** | **<0.001** |
| Joint pain | 2.091 | 0.400 | **8.1 (3.7-17.7)** | **<0.001** |
| Fever | 1.353 | 0.382 | **3.9 (1.8-8.2)** | **<0.001** |
| Chills | 1.697 | 0.416 | **5.5 (2.4-12.3)** | **<0.001** |
| Cough | 1.550 | 0.690 | **4.7 (1.2-18.2)** | **0.025** |
| Difficulty in breathing or Shortness of breath | 2.708 | 0.769 | **15.0 (3.3-67.8)** | **<0.001** |
| Nausea/vomiting | 2.831 | 1.055 | **17.0 (2.1-134.0)** | **0.007** |
| Headache | 1.666 | 0.377 | **5.3 (2.5-11.1)** | **<0.001** |
| Rash | 1.137 | 0.404 | **3.1 (1.4-6.9)** | **0.005** |
| Fatigue | 1.799 | 0.325 | **6.0 (3.2-11.4)** | **<0.001** |
| Diarrhoea | 1.853 | 0.808 | **6.4 (1.3-31.1)** | **0.022** |
| Abdominal pain | 1.529 | 0.682 | **4.6 (1.2-17.6)** | **0.025** |
| High pulse rate or palpitations | 1.727 | 0.540 | **5.6 (2.0-16.2)** | **0.001** |
| Rise in blood pressure | 1.709 | 0.809 | **5.5 (1.1-26.9)** | **0.035** |
| Dizziness | 1.292 | 0.470 | **3.6 (1.4-9.2)** | **0.006** |
| Weakness and tingling in the feet and legs | 1.052 | 0.465 | **2.9 (1.2-7.1)** | **0.024** |
| Pricking or pins and needles sensations in the hands and feet | 1.318 | 0.625 | **3.7 (1.1-12.7)** | **0.035** |
| **Major symptoms** | 0.763 | 0.286 | **2.1 (1.2-3.8)** | **0.008** |
| Marked difficulty in breathing | 1.055 | 0.523 | **2.9 (1.0-8.0)** | **0.044** |
| Throat closure | 1.818 | 0.825 | **6.2 (1.2-31.0)** | **0.027** |

**Supplementary Table 9 a. AEs distribution according to the vaccines in IIM group (IBM excluded)**

|  | **IIM (IBM-)** | | **SAIDs** | | **HC** | | **OR1 (95%CI)** | **OR2**  **(95%CI)** | **p1** | **p2** |
| --- | --- | --- | --- | --- | --- | --- | --- | --- | --- | --- |
|  | **N (1141)** | **%**  **(100)** | **N (4432)** | **%**  **(100)** | **N (2937)** | **%**  **(100)** |  |  |  |  |
| **Minor AEs** | 209 | 18.3 | 948 | 21.4 | 561 | 19.1 | 0.8 (0.7-1.0) |  | 0.022 | 0.566 |
| Injection site (arm) pain and soreness | 107 | 9.4 | 558 | 12.6 | 365 | 12.4 | 0.7 (0.6-0.9) | 0.7 (0.6-0.9) | 0.003 | 0.006 |
| Myalgia | 98 | 8.6 | 443 | 10.0 | 217 | 7.4 |  |  | 0.151 | 0.197 |
| Body ache | 102 | 8.9 | 488 | 11.0 | 238 | 8.1 | 0.8 (0.6-1.0) |  | 0.042 | 0.386 |
| Joint pain | 88 | 7.7 | 486 | 11.0 | 165 | 5.6 | 0.7 (0.5-0.9)# | 1.4 (1.1-1.8) | 0.001 | 0.013 |
| Fever | 67 | 5.9 | 359 | 8.1 | 248 | 8.4 | 0.7 (0.5-0.9) | 0.7 (0.5-0.9) | 0.011 | 0.006 |
| Chills | 68 | 6.0 | 285 | 6.4 | 162 | 5.5 |  |  | 0.558 | 0.581 |
| Cough | 23 | 2.0 | 111 | 2.5 | 54 | 1.8 |  |  | 0.336 | 0.709 |
| Difficulty in breathing or Shortness of breath | 34 | 3.0 | 126 | 2.8 | 58 | 2.0 |  |  | 0.807 | 0.052 |
| Nausea/vomiting | 28 | 2.5 | 171 | 3.9 | 45 | 1.5 | 0.6 (0.4-0.9) | 1.6 (1.0-2.6) | 0.022 | 0.046 |
| Headache | 82 | 7.2 | 428 | 9.7 | 193 | 6.6 | 0.7 (0.6-0.9)# |  | 0.010 | 0.482 |
| Rash | 54 | 4.7 | 129 | 2.9 | 27 | 0.9 | 1.7 (1.1-2.3)# | 5.4 (3.4-8.5) | 0.002 | <.001 |
| Fatigue | 113 | 9.9 | 507 | 11.4 | 198 | 6.7 |  | 1.5 (1.2-1.9) | 0.140 | 0.001 |
| Diarrhoea | 25 | 2.2 | 117 | 2.6 | 42 | 1.4 |  |  | 0.390 | 0.086 |
| Abdominal pain | 23 | 2.0 | 101 | 2.3 | 33 | 1.1 |  | 1.8 (1.1-3.1) | 0.590 | 0.028 |
| High pulse rate or palpitations | 35 | 3.1 | 167 | 3.8 | 73 | 2.5 |  |  | 0.258 | 0.299 |
| Rise in blood pressure | 17 | 1.5 | 86 | 1.9 | 30 | 1.0 |  |  | 0.313 | 0.208 |
| Fainting | 4 | 0.4 | 22 | 0.5 | 12 | 0.4 |  |  | 0.519 | 0.790 |
| Dizziness | 40 | 3.5 | 221 | 5.0 | 68 | 2.3 | 0.7 (0.5-1.0) | 1.5 (1.0-2.3) | 0.035 | 0.034 |
| Chest pain | 15 | 1.3 | 120 | 2.7 | 30 | 1.0 | 0.5 (0.3-0.8) |  | 0.006 | 0.421 |
| Swelling in the extremities | 20 | 1.8 | 100 | 2.3 | 29 | 1.0 |  | 1.8 (1.0-3.2) | 0.295 | 0.044 |
| Weakness and tingling in the feet and legs | 46 | 4.0 | 166 | 3.7 | 65 | 2.2 |  | 1.9 (1.3-2.7) | 0.654 | 0.001 |
| Pricking or pins and needles sensations in the hands and feet | 35 | 3.1 | 137 | 3.1 | 42 | 1.4 |  | 2.2 (1.4-3.4) | 0.965 | 0.001 |
| Visual disturbances (loss of vision, blurring of vision, etc.) | 16 | 1.4 | 115 | 2.6 | 28 | 1.0 | 0.5 (0.3-0.9)# |  | 0.018 | 0.213 |
| Bleeding/bruising on the body | 13 | 1.1 | 67 | 1.5 | 15 | 0.5 |  | 2.2 (1.1-4.7) | 0.345 | 0.029 |
| Petechial rash | 11 | 1.0 | 54 | 1.2 | 11 | 0.4 |  | 2.6 (1.1-6.0) | 0.475 | 0.021 |
| **Major AEs** | 129 | 11.3 | 685 | 15.5 | 375 | 12.8 | 0.7 (0.6-0.9) |  | <0.001 | 0.203 |
| Anaphylaxis | 17 | 1.5 | 66 | 1.5 | 47 | 1.6 |  |  | 1.000 | 0.799 |
| Marked difficulty in breathing | 42 | 3.7 | 135 | 3.0 | 77 | 2.6 |  |  | 0.277 | 0.071 |
| Throat closure | 20 | 1.8 | 63 | 1.4 | 38 | 1.3 |  |  | 0.377 | 0.266 |
| Severe rashes | 37 | 3.2 | 108 | 2.4 | 54 | 1.8 |  | 1.8 (1.2-2.7) | 0.128 | 0.006 |
| **Hospitalisation** | 37 | 3.2 | 201 | 4.5 | 77 | 2.6 |  |  | 0.058 | 0.280 |

**Supplementary Table 9 b. Effects of COVID-19 vaccination in patients with IIMs (IBM excluded) vs other SAIDs and HCs**

|  | **BNT162b2 (Pfizer)** | | **ChadOx1 nCOV-19 (Oxford/ AstraZeneca)** | | **MRNA-1273 (Moderna)** | | **ChAdOx1 nCoV-19 (Covishield Serum Institute India)** | | **Sinovac-CoronaVac** | |
| --- | --- | --- | --- | --- | --- | --- | --- | --- | --- | --- |
|  | **N (727)** | **% (100)** | **N (126)** | **% (100)** | **N (451)** | **% (100)** | **N (14)** | **% (100)** | **N (28)** | **% (100)** |
| **Minor AEs** | 123 | 16.9 | 31 | 24.6 | 78 | 17.3 | 4 | 28.6 | 9 | 32.1 |
| Injection site (arm) pain and soreness | 62 | 8.5 | **19** | **15.1*#** | 48 | 10.6 | 1 | 7.1 | **6** | **21.4*** |
| Myalgia | 57 | 7.8 | 16 | 12.7 | 40 | 8.9 | 1 | 7.1 | 5 | 17.9 |
| Body ache | 56 | 7.7 | **18** | **14.3*#** | 41 | 9.1 | 2 | 14.3 | **6** | **21.4*** |
| Joint pain | 49 | 6.7 | **17** | **13.5**#** | 39 | 8.6 | 2 | 14.3 | **5** | **17.9*** |
| Fever | 38 | 5.2 | **13** | **10.3*** | 29 | 6.4 | 2 | 14.3 | **5** | **17.9**** |
| Chills | 41 | 5.6 | 12 | 9.5 | 26 | 5.8 | 1 | 7.1 | 3 | 10.7 |
| Cough | 13 | 1.8 | 1 | 0.8 | 6 | 1.3 | 1 | 7.1 | **3** | **10.7**#** |
| Difficulty in breathing or Shortness of breath | 20 | 2.8 | 4 | 3.2 | 13 | 2.9 | **2** | **14.3*** | 2 | 7.1 |
| Nausea/vomiting | ***12*** | ***1.7*#*** | 5 | 4.0 | 15 | 3.3 | 0 | 0 | 0 | 0 |
| Headache | 53 | 7.3 | 14 | 11.1 | 26 | 5.8 | 0 | 0 | 3 | 10.7 |
| Rash | ***23*** | ***3.2**#*** | 3 | 2.4 | 25 | 5.5 | **3** | **21.4**** | 2 | 7.1 |
| Fatigue | 66 | 9.1 | 18 | 14.3 | 41 | 9.1 | 2 | 14.3 | 4 | 14.3 |
| Diarrhoea | 16 | 2.2 | **6** | **4.8*** | 9 | 2.0 | 1 | 7.1 | 1 | 3.6 |
| Abdominal pain | ***9*** | ***1.2*#*** | 4 | 3.2 | 9 | 2.0 | 1 | 7.1 | **3** | **10.7**** |
| High pulse rate or palpitations | 24 | 3.3 | 5 | 4.0 | 10 | 2.2 | **2** | **14.3*** | **4** | **14.3***** |
| Rise in blood pressure | 12 | 1.7 | 4 | 3.2 | 5 | 1.1 | 0 | 0 | 1 | 3.6 |
| Fainting | 2 | 0.3 | **2** | **1.6*** | 2 | 0.4 | **1** | **7.1***** | **2** | **7.1***** |
| Dizziness | 26 | 3.6 | 8 | 6.3 | 15 | 3.3 | 1 | 7.1 | **4** | **14.3**#** |
| Chest pain | 9 | 1.2 | 3 | 2.4 | 7 | 1.6 | 0 | 0 | 1 | 3.6 |
| Swelling in the extremities | 12 | 1.7 | **5** | **2.2*#** | 7 | 1.6 | 1 | 7.1 | **2** | **7.1*** |
| Weakness and tingling in the feet and legs | 26 | 3.6 | **10** | **7.9*#** | 14 | 3.1 | 1 | 7.1 | **4** | **14.3*#** |
| Pricking or pins and needles sensations in the hands and feet | 20 | 2.8 | **8** | **6.3*#** | 9 | 2.0 | 1 | 7.1 | **5** | **17.9***#** |
| Visual disturbances (loss of vision, blurring of vision, etc.) | 9 | 1.2 | **5** | **4.0**#** | 7 | 1.6 | 1 | 7.1 | **2** | **7.1*#** |
| Bleeding/bruising on the body | 9 | 1.2 | **5** | **4.0**#** | 2 | 0.4 | **1** | **7.1*** | **2** | **7.1**** |
| Petechial rash | ***3*** | ***0.4*#*** | 2 | 1.6 | 6 | 1.3 | **1** | **7.1*** | **2** | **7.1**#** |
| **Major AEs** | ***64*** | ***8.8***#*** | 19 | 15.1 | 56 | 12.4 | **7** | **50***** | **10** | **35.7***#** |
| Anaphylaxis | 11 | 1.5 | 4 | 3.2 | 9 | 2.0 | **3** | **21.4***** | **5** | **17.9***#** |
| Marked difficulty in breathing | 24 | 3.3 | 7 | 5.6 | 15 | 3.3 | **5** | **35.7***** | **4** | **14.3**** |
| Throat closure | 11 | 1.5 | **5** | **4.0*** | **13** | **2.9*#** | **3** | **21.4***** | **5** | **17.9***#** |
| Severe rashes | 18 | 2.5 | 5 | 4.0 | 19 | 4.2 | **4** | **28.6***** | **6** | **21.4***#** |
| **Hospitalisation** | 20 | 2.8 | **8** | **6.3*** | 18 | 4.0 | **4** | **28.6***** | **5** | **17.9***** |

Bold indicates increased odds ratio vs the remaining vaccines. Bold + underlined indicates decreased odds ratio vs remaining vaccines. Abbreviations: AE, adverse event; AID, autoimmune disease; HC, healthy control; IIM, idiopathic inflammatory myopathy.

#Significant according to binary logistic regression adjusted for age, gender, ethnicity, and immunosuppressant dose, and stratified by country. *P < .05, **P < .005, ***P < .001.

**Supplementary Table 9 c. Factors significant in multivariable analysis (BLR) between IIMs and SAIDs and between different vaccines (IBM excluded)**

| *IIMs as compared to other SAIDs (adjusted for age, gender, ethnicity, comorbidity, number of vaccine doses received, IS drugs, and stratified for country of origin)* | | | | |
| --- | --- | --- | --- | --- |
| Joints pain | -0.380 | 0.133 | **0.7 (0.5-0.9)** | **0.004** |
| Headache | -0.375 | 0.139 | **0.7 (0.5-0.9)** | **0.007** |
| Rash | 0.566 | 0.194 | **1.8 (1.2-2.6)** | **0.004** |
| Visual disturbances (loss of vision, blurring of vision, etc.) | -0.710 | 0.301 | **0.5 (0.3-0.9)** | **0.018** |
| *BNT162b2 (Pfizer) vs rest of vaccines in IIM patients IBM excluded (adjusted for age, gender, ethnicity, comorbidity, dose of immunosuppressant, and stratified for country of origin)* | | | | |
| Nausea/vomiting | -0.868 | 0.405 | **0.4 (0.2-0.9)** | **0.032** |
| Rash | -0.783 | 0.300 | **0.5 (0.3-0.8)** | **0.009** |
| Abdominal pain | -1.030 | 0.445 | **0.4 (0.1-0.9)** | **0.021** |
| Petechial rash | -1.593 | 0.701 | **0.2 (0.1-0.8)** | **0.023** |
| Major ADEs | -0.701 | 0.202 | **0.5 (0.3-0.7)** | **0.001** |
| *ChadOx1 nCOV-19 (Oxford/ AstraZeneca)* *vs rest of vaccines in IIM patients IBM excluded (adjusted for age, gender, ethnicity, comorbidity, dose of immunosuppressant, and stratified for country of origin)* | | | | |
| Injection site (arm) pain and soreness | 0.640 | 0.290 | **1.9 (1.1-3.3)** | **0.027** |
| Body ache | 0.618 | 0.294 | **1.9 (1.0-3.3)** | **0.035** |
| Joint pain | 0.838 | 0.304 | **2.3 (1.3-4.2)** | **0.006** |
| Swelling in the extremities | 1.283 | 0.564 | **3.6 (1.2-10.9)** | **0.023** |
| Weakness and tingling in the feet and legs | 0.987 | 0.394 | **2.7 (1.2-5.8)** | **0.012** |
| Pricking or pins and needles sensations in the hands and feet | 1.072 | 0.451 | **2.9 (1.2-7.1)** | **0.017** |
| Visual disturbances (loss of vision, blurring of vision, etc.) | 1.757 | 0.591 | **5.8 (1.8-18,5)** | **0.003** |
| Bleeding/bruising on the body | 1.720 | 0.652 | **5.6 (1.6-20.1)** | **0.008** |
| *MRNA-1273 (Moderna) vs rest of vaccines in IIM patients IBM excluded (adjusted for age, gender, ethnicity, comorbidity, dose of immunosuppressant, and stratified for country of origin)* | | | | |
| Throat closure | 1.145 | 0.520 | **3.1 (1.1-8.7)** | **0.028** |
| *Sinovac-CoronaVac vs rest of vaccines in IIM patients IBM excluded (adjusted for age, gender, ethnicity, comorbidity, dose of immunosuppressant, and stratified for country of origin)* | | | | |
| Cough | 1.786 | 0.815 | **6.0 (1.2-29.5)** | **0.028** |
| Dizziness | 1.512 | 0.688 | **4.5 (1.2-17.5)** | **0.028** |
| Weakness and tingling in the feet and legs | 1.387 | 0.667 | **4.0 (1.1-14.8)** | **0.038** |
| Pricking or pins and needles sensations in the hands and feet | 1.952 | 0.679 | **7.0 (1.9-26.7)** | **0.004** |
| Visual disturbances (loss of vision, blurring of vision, etc.) | 2.288 | 0.977 | **9.9 (1.5-66.8)** | **0.019** |
| Petechial rash | 2.208 | 1.120 | **9.1 (1.0-81.7)** | **0.049** |
| **Major AEs** | 0.989 | 0.475 | **2.7 (1.1-6.8)** | **0.037** |
| Anaphylaxis | 2.245 | 0.775 | **9.4 (2.1-43.1)** | **0.004** |
| Throat closure | 1.923 | 0.738 | **6.8 (1.6-29.0)** | **0.009** |
| Severe rashes | 1.709 | 0.655 | **5.5 (1.5-19.9)** | **0.009** |

**Supplementary Table 10 a. AEs distribution according to the vaccines in IIM group (symptoms duration more than 30 days)**

|  | **IIM** | | | **SAIDs** | | **HC** | | **OR1 (95%CI)** | **OR2**  **(95%CI)** | **p1** | **p2** |
| --- | --- | --- | --- | --- | --- | --- | --- | --- | --- | --- | --- |
|  | **N (95)** | **%**  **(100)** | **N (296)** | | **%**  **(100)** | **N (91)** | **%**  **(100)** |  |  |  |  |
| **Minor AEs** | 86 | 90.5 | 272 | | 91.9 | 88 | 96.7 |  |  | 0.677 | 0.087 |
| Injection site (arm) pain and soreness | 41 | 43.2 | 144 | | 48.6 | 40 | 44.0 |  |  | 0.351 | 0.913 |
| Myalgia | 48 | 50.5 | 146 | | 49.3 | 34 | 37.4 |  |  | 0.838 | 0.071 |
| Body ache | 47 | 49.5 | 156 | | 52.7 | 33 | 36.3 |  |  | 0.584 | 0.069 |
| Joint pain | 43 | 45.3 | 185 | | 62.5 | 34 | 37.4 | 0.5 (0.3-0.8)# |  | 0.003 | 0.274 |
| Fever | 27 | 28.4 | 73 | | 24.7 | 17 | 18.7 |  |  | 0.465 | 0.118 |
| Chills | 26 | 27.4 | 77 | | 26.0 | 17 | 18.7 |  |  | 0.794 | 0.160 |
| Cough | 12 | 12.6 | 37 | | 12.5 | 7 | 7.7 |  |  | 0.973 | 0.266 |
| Difficulty in breathing or Shortness of breath | 23 | 24.2 | 40 | | 13.5 | 13 | 14.3 | 2.0 (1.2-3.6)# |  | 0.014 | 0.087 |
| Nausea/vomiting | 10 | 10.5 | 46 | | 15.5 | 9 | 9.9 |  |  | 0.225 | 0.886 |
| Headache | 35 | 36.8 | 125 | | 42.2 | 37 | 40.7 |  |  | 0.353 | 0.593 |
| Rash | 29 | 30.5 | 42 | | 14.2 | 4 | 4.4 | 2.7 (1.5-4.6)# | 9.6 (3.2-28.5)# | <.001 | <.001 |
| Fatigue | 61 | 64.2 | 172 | | 58.1 | 47 | 51.6 |  |  | 0.292 | 0.083 |
| Diarrhoea | 14 | 14.7 | 34 | | 11.5 | 9 | 9.9 |  |  | 0.401 | 0.315 |
| Abdominal pain | 13 | 13.7 | 29 | | 9.8 | 9 | 9.9 |  |  | 0.287 | 0.423 |
| High pulse rate or palpitations | 19 | 20.0 | 62 | | 20.9 | 22 | 24.2 |  |  | 0.843 | 0.492 |
| Rise in blood pressure | 8 | 8.4 | 33 | | 11.1 | 10 | 11.0 |  |  | 0.450 | 0.554 |
| Fainting | 2 | 2.1 | 7 | | 2.4 | 2 | 2.2 |  |  | 0.883 | 0.965 |
| Dizziness | 24 | 25.3 | 76 | | 25.7 | 14 | 15.4 |  |  | 0.936 | 0.095 |
| Chest pain | 10 | 10.5 | 46 | | 15.5 | 9 | 9.3 |  |  | 0.225 | 0.886 |
| Swelling in the extremities | 9 | 9.5 | 43 | | 14.5 | 8 | 8.8 |  |  | 0.207 | 0.872 |
| Weakness and tingling in the feet and legs | 23 | 24.2 | 59 | | 19.9 | 16 | 17.6 |  |  | 0.373 | 0.267 |
| Pricking or pins and needles sensations in the hands and feet | 17 | 17.9 | 54 | | 18.2 | 16 | 17.6 |  |  | 0.939 | 0.956 |
| Visual disturbances (loss of vision, blurring of vision, etc.) | 10 | 10.5 | 45 | | 15.2 | 8 | 8.8 |  |  | 0.254 | 0.689 |
| Bleeding/bruising on the body | 8 | 8.4 | 25 | | 8.4 | 4 | 4.4 |  |  | 0.994 | 0.264 |
| Petechial rash | 7 | 7.4 | 20 | | 6.8 | 2 | 2.2 |  |  | 0.838 | 0.100 |
| **Major AEs** | 34 | 35.8 | 109 | | 36.8 | 24 | 26.4 |  |  | 0.855 | 0.166 |
| Anaphylaxis | 6 | 6.3 | 11 | | 3.7 | 3 | 3.3 |  |  | 0.280 | 0.337 |
| Marked difficulty in breathing | 15 | 15.8 | 27 | | 9.1 | 6 | 6.6 |  | 2.7 (1.0-7.2) | 0.068 | 0.048 |
| Throat closure | 7 | 7.4 | 12 | | 4.1 | 2 | 2.2 |  |  | 0.191 | 0.100 |
| Severe rashes | 13 | 13.7 | 24 | | 8.1 | 5 | 5.5 |  |  | 0.106 | 0.059 |
| **Hospitalisation** | 10 | 10.5 | 25 | | 8.4 | 8 | 8.8 |  |  | 0.537 | 0.689 |

**Supplementary Table 10 b. Factors significant in multivariable analysis (BLR) between IIM and HCs, IIMs and SAIDs (symptoms duration more than 30 days)**

| *IIMs as compared to HCs (adjusted for age, gender, ethnicity, comorbidity)** | | | | |
| --- | --- | --- | --- | --- |
|  | B coefficient | S.E. | Exp (B) CI (95%) | P value |
| Rash | 2.321 | 0.668 | 10.2 (2.8-37.7) | 0.001 |
| Marked difficulty in breathing | 1.463 | 0.700 | 4.3 (1.1-17.0) | 0.037 |
| *IIMs as compared to other SAIDs (adjusted for age, gender, ethnicity, comorbidity, dose of IS)* | | | | |
| Joints pain | -0.874 | 0.270 | **0.4 (0.2-0.7)** | **0.001** |
| Shortness of breath | 0.921 | 0.343 | **2.5 (1.3-4.9)** | **0.007** |
| Rash | 1.004 | 0.327 | **2.7 (1.4-5.2)** | **0.002** |

* After adjustment for IS drugs prescription no statistically significant differences between IIMs and HCs were observed.

**Supplementary Table 11. Distribution of respondents according to place of origin**

| Country of Residence | Responses | | |
| --- | --- | --- | --- |
|  | Absolute number | % |  |
| United States of America | 1153 | 13.16 |  |
| United Kingdom of Great Britain and Northern Ireland | 943 | 10.76 |  |
| Mexico | 853 | 9.74 |  |
| India | 482 | 5.50 |  |
| Thailand | 384 | 4.38 |  |
| Italy | 370 | 4.22 |  |
| Colombia | 214 | 2.44 |  |
| Guatemala | 213 | 2.43 |  |
| Brazil | 207 | 2.36 |  |
| Peru | 207 | 2.36 |  |
| Egypt | 177 | 2.02 |  |
| Poland | 176 | 2.01 |  |
| Taiwan | 168 | 1.92 |  |
| Nepal | 162 | 1.85 |  |
| Malaysia | 160 | 1.83 |  |
| Bangladesh | 155 | 1.77 |  |
| Spain | 148 | 1.69 |  |
| Bulgaria | 143 | 1.63 |  |
| Chile | 136 | 1.55 |  |
| Canada | 124 | 1.42 |  |
| Panama | 121 | 1.38 |  |
| Australia | 119 | 1.36 |  |
| Saudi Arabia | 117 | 1.34 |  |
| Ecuador | 111 | 1.27 |  |
| Japan | 97 | 1.11 |  |
| Turkey | 95 | 1.08 |  |
| Lebanon | 93 | 1.06 |  |
| Pakistan | 91 | 1.04 |  |
| Argentina | 84 | 0.96 |  |
| Ghana | 83 | 0.95 |  |
| Philippines | 79 | 0.90 |  |
| Morocco | 78 | 0.89 |  |
| Costa Rica | 78 | 0.89 |  |
| Sweden | 76 | 0.87 |  |
| Venezuela | 75 | 0.86 |  |
| France | 71 | 0.81 |  |
| Nigeria | 70 | 0.80 |  |
| Germany | 66 | 0.75 |  |
| United Arab Emirates | 60 | 0.68 |  |
| Russian Federation | 56 | 0.64 |  |
| Hungary | 55 | 0.63 |  |
| Dominican Republic | 48 | 0.55 |  |
| Bolivia | 29 | 0.33 |  |
| Iraq | 29 | 0.33 |  |
| Mauritius | 28 | 0.32 |  |
| Portugal | 28 | 0.32 |  |
| Jordan | 22 | 0.25 |  |
| Kuwait | 22 | 0.25 |  |
| Ireland | 20 | 0.23 |  |
| Paraguay | 18 | 0.21 |  |
| Switzerland | 17 | 0.19 |  |
| Oman | 13 | 0.15 |  |
| Austria | 10 | 0.11 |  |
| Algeria | 9 | 0.10 |  |
| Netherlands | 9 | 0.10 |  |
| Bahrain | 6 | 0.07 |  |
| Honduras | 6 | 0.07 |  |
| China | 5 | 0.06 |  |
| South Africa | 4 | 0.05 |  |
| Finland | 4 | 0.05 |  |
| Nicaragua | 4 | 0.05 |  |
| Ukraine | 4 | 0.05 |  |
| Ethiopia | 4 | 0.05 |  |
| El Salvador | 4 | 0.05 |  |
| Romania | 3 | 0.03 |  |
| Belgium | 3 | 0.03 |  |
| Malta | 3 | 0.03 |  |
| Afghanistan | 3 | 0.03 |  |
| Israel | 2 | 0.02 |  |
| Syrian Arab Republic | 2 | 0.02 |  |
| Armenia | 2 | 0.02 |  |
| Angola | 2 | 0.02 |  |
| Vanuatu | 2 | 0.02 |  |
| Czech Republic | 2 | 0.02 |  |
| Bahamas | 2 | 0.02 |  |
| Haiti | 2 | 0.02 |  |
| New Zealand | 2 | 0.02 |  |
| Qatar | 2 | 0.02 |  |
| Andorra | 2 | 0.02 |  |
| Cyprus | 2 | 0.02 |  |
| Libya | 2 | 0.02 |  |
| Zimbabwe | 1 | 0.01 |  |
| Iceland | 1 | 0.01 |  |
| Tajikistan | 1 | 0.01 |  |
| Greece | 1 | 0.01 |  |
| Malawi | 1 | 0.01 |  |
| Central African Republic | 1 | 0.01 |  |
| Cameroon | 1 | 0.01 |  |
| Sao Tome and Principe | 1 | 0.01 |  |
| Jamaica | 1 | 0.01 |  |
| Trinidad and Tobago | 1 | 0.01 |  |
| Lao Peopleâ€™s Democratic Republic | 1 | 0.01 |  |
| Sudan | 1 | 0.01 |  |
| Indonesia | 1 | 0.01 |  |
| Albania | 1 | 0.01 |  |
| Eritrea | 1 | 0.01 |  |
| Somalia | 1 | 0.01 |  |
| Tuvalu | 1 | 0.01 |  |
|  | 1 | 0.01 |  |
| Azerbaijan | 1 | 0.01 |  |
| Croatia | 1 | 0.01 |  |
| Uzbekistan | 1 | 0.01 |  |
| Burkina Faso | 1 | 0.01 |  |
| Kyrgyzstan | 1 | 0.01 |  |
| Liechtenstein | 1 | 0.01 |  |
| Luxembourg | 1 | 0.01 |  |
| Serbia | 1 | 0.01 |  |
| Singapore | 1 | 0.01 |  |
| State of Palestine | 1 | 0.01 |  |
| Uruguay | 1 | 0.01 |  |

| Supplemental Table 12a. Characteristics of patients with IIMs requiring hospitalization (with vaccination as a most probable cause) | | | | | | | | | |
| --- | --- | --- | --- | --- | --- | --- | --- | --- | --- |
| **S.No.** | **Age** | **Sex** | **Ethnicity** | **IIM subtype** | **Vaccine received** | **Reason for Hospitalization** | **Associated symptoms** | **IS therapy pre-vaccination** | **Comorbidities** |
| 1 | 52 | F | Hispanic | Myositis/ ASSD | Moderna | - Sudden onset quadriparesis - Severe weakness and fatigue | - Injection site pain - Myalgia, body ache, join pain, fever, chills, headache - Difficulty in breathing/shortness of breath - Nausea/vomiting, abdominal pain - Rash, severe diffuse body rash (hives) - Chest pain, rise in BP, tachycardia, palpitations - Weakness and tingling sensation in feet and legs - Tongue swelling/throat closure | None | HTN |
| 2 | 64 | F | Caucasian | Myositis/ ASSD | Oxford/AstraZeneca | - Severe weakness - Elevated creatinine level | - Myalgia | None | None |
| 3 | 51 | F | Caucasian | Myositis/ ASSD | Pfizer-BioNTech | - Severe fatigue, weakness, and inability to walk. - Elevation in creatine kinase levels | - Injection site pain - Myalgia, headache, fatigue - Visual disturbances - Weakness and tingling sensation in feet and legs - Pricking/pins and needles sensation in hands and feet | None | T2DM |
| 4 | 55 | M | Hispanic | Myositis/ ASSD | Moderna | - Interstitial myositis | - Injection site pain - Myalgia, body ache, fatigue - Tachycardia, palpitations | None | None |
| 5 | 61 | M | Caucasian | Polymyositis | Pfizer-BioNTech | - Difficulty in breathing/ shortness of breath | - Marked difficulty in breathing | Methotrexate | T2DM, ILD |
| 6 | 54 | F | Caucasian | OM | Moderna | - Deep vein thrombosis | None | Glucocorticoids | Hemolytic anemia/ idiopathic thrombocytopenic purpura (ITP), Thyroid disorder (Hypo/Hyperthyroidism |
| 7 | 66 | F | Caucasian | Myositis/ ASSD | Moderna | - Severe nausea, vertigo - Severe dehydration | - Injection site pain - Myalgia, fatigue, body ache, fever, joint pain | None | Asthma |
| 8 | 36 | M | Caucasian | Myositis/ ASSD | Moderna | - Severe myalgia - Elevation in creatine kinase levels (22,000) | None | Azathioprine + IVIg/scIg + Glucocorticoids | Hyperlipidemia |
| 9 | 38 | F | Caucasian | Myositis/ ASSD | Pfizer-BioNTech | - Flare of autoimmune disease | - Injection site pain - Myalgia, body ache, fever, joint pain - Tachycardia, palpitations - Peripheral edema - Cough | None | None |
| 10 | 65 | F | Caucasian | Myositis/ ASSD | Pfizer-BioNTech | - Dyspnea | - Fever | Rituximab | ILD, hyperlipidemia |

IIM: idiopathic inflammatory myopathies; IS: immunosuppressive; F: female; M: male; SLE: systemic lupus erythematosus; MMF: mycophenolate mofetil; HCQ: hydroxychloroquine; IVIg/scIg: intravenous immunoglobulin/subcutaneous immunoglobulin; CKD: chronic kidney disease; HC: healthy control; ASSD: antisynthetase syndrome; HTN: hypertension; T2DM: type 2 diabetes mellitus; ILD: interstitial lung disease; TNF: tumour necrosis factor; OM: Overlap myositis with SLE or Sjogren’s syndrome or systemic sclerosis or rheumatoid arthritis; MCTD: mixed connective tissue disease

| Supplemental Table 12b. Characteristics of non-IIM respondents (SAIDs and HCs) requiring hospitalization (with vaccination as a most probable cause) | | | | | | | | | |
| --- | --- | --- | --- | --- | --- | --- | --- | --- | --- |
| **S.No.** | **Age** | **Sex** | **Ethnicity** | **SAID**  **diagnosis** | **Vaccine received** | **Reason for Hospitalization** | **Associated symptoms** | **IS therapy pre-vaccination** | **Comorbidities** |
| 1 | 51 | F | Hispanic | Rheumatoid arthritis | Sinovac | - Severe generalized body pain - High grade fever | None | Methotrexate + Rituximab | None |
| 2 | 65 | M | Caucasian | Polymyalgia rheumatica | Oxford/AstraZeneca | - Herpes Zoster Ophthalmicus - Severe headache and retro-orbital pain | Minor non-specific symptoms | Prednisolone | None |
| 3 | 39 | F | Hispanic | SLE | Moderna | - Generalized lymphadenopathy - Leg swelling (fluid retention)- Probable Deep venous thrombosis | - Injection site pain - Fever - Headache - Peripheral edema - Visual disturbances - Bleeding/bruising on the body - Petechial rash | MMF + HCQ + oral Tacrolimus + IVIg/scIg | CKD |
| 4 | 50 | F | Caucasian | SLE | Oxford/AstraZeneca | - Arrythmia - Severe SLE flare (chest pain, fatigue and increase in typical SLE symptoms) - Suspected Endocarditis | - Myalgia, Fatigue, and Joint pain - Cough, difficulty in breathing/shortness of breath - Rash - Chest pain - Swollen glands - Tachycardia, Palpitations - Mouth ulcers | MMF + HCQ + Rituximab | CKD |
| 5 | 43 | M | Do not wish to disclose | Rheumatoid arthritis | Pfizer-BioNTech | - High grade fever | - Chills - Difficulty in breathing/shortness of breath - Dizziness - Peripheral edema | Golimumab | None |
| 6 | 38 | F | Hispanic | None (HC) | Pfizer-BioNTech | - Guillain-Barré syndrome | - Headache | None | Thyroid disorder (Hypo/Hyperthyroidism |
| 7 | 61 | F | Caucasian | Rheumatoid arthritis | Pfizer-BioNTech | - Severe diffuse body rash (15 days post COVID-19 vaccine) -Reaction to COVID-19 vaccine confirmed by 2 punch biopsies | - Petechial rash | None | Asthma, T2DM |
| 8 | 50 | F | Caucasian | SLE | Oxford/AstraZeneca | - Arrythmia - Myocarditis | - Chest pain, tachycardia, palpitations - Dizziness - Visual disturbances - Cough - Joint pain | MMF + HCQ + Rituximab | None |
| 9 | 36 | M | Caucasian | Ankylosing spondylitis | Johnson & Johnson (J&J) | - Vasculitis | - Myalgia, body ache, joint pain, Fever, Fatigue - Tachycardia, palpitations, rise in BP - Peripheral edema - Weakness and tingling sensation in feet and legs - Pricking/pins and needles sensation in hands and feet | None | None |
| 10 | 51 | F | Caucasian | Systemic sclerosis | Pfizer-BioNTech | - Cardiac problems | - Injection site pain - Myalgia, headache, fatigue, body ache - Dizziness - Chest pain, tachycardia, palpitations, rise in BP - Difficulty in breathing/shortness of breath - Peripheral edema | None | Hemolytic anemia/ idiopathic thrombocytopenic purpura (ITP), Thyroid disorder (Hypo/Hyperthyroidism |
| 11 | 30 | F | Asian | SLE | Oxford/AstraZeneca | - Vomiting (for 3 days) | None | HCQ + Prednisolone | None |
| 12 | 62 | F | Caucasian | Psoriatic arthritis | Pfizer-BioNTech | - Difficulty in breathing/shortness of breath - Fainting | - Myalgia, headache, fatigue, body ache, join pain, chills, fatigue - Chest pain, tachycardia, palpitations, rise in BP - Dizziness - Peripheral edema - Weakness and tingling sensation in feet and legs - Pricking/pins and needles sensation in hands and feet - Visual disturbances | Local corticosteroid cream | None |
| 13 | 64 | F | Asian | Rheumatoid arthritis | Oxford/AstraZeneca | - Dizziness, Fainting - Nausea/Vomiting | - Injection site pain - Myalgia, headache, fatigue, body ache - Difficulty in breathing/shortness of breath - Tachycardia, palpitations, rise in BP - Visual disturbances - Weakness and tingling sensation in feet and legs | None | None |
| 14 | 51 | F | Hispanic | Thyroid disorder (Hypo/Hyperthyroidism | Oxford/AstraZeneca | - Hypoglycemia | None | Cabergoline + Fluoxetine | Fibromyalgia |
| 15 | 38 | M | Caucasian | Psoriatic arthritis | Pfizer-BioNTech | - Anaphylaxis | - Marked difficulty in breathing - Tongue swelling/throat closure - Severe diffuse body rash (hives) | Methotrexate + Leflunomide + Anti-TNF agent | Hyperlipidemia, Arrythmia |
| 16 | 38 | F | Hispanic | Rheumatoid arthritis | Pfizer-BioNTech | - Dyspnea, Tachycardia - Vomiting, Abdominal pain - Malaise | - Injection site pain - Myalgia, headache, fatigue, body ache, fever, chills | None | Thyroid disorder (Hypo/Hyperthyroidism |
| 17 | 54 | F | Caucasian | OM | Moderna | - Deep vein thrombosis | None | Glucocorticoids | Hemolytic anemia/ idiopathic thrombocytopenic purpura (ITP), Thyroid disorder (Hypo/Hyperthyroidism |
| 18 | 43 | F | Caucasian | Vasculitis | Pfizer-BioNTech | - Pulmonary haemorrhage - Pericarditis - Episcleritis - Severe joint pain and inability to walk | - Injection site pain - Myalgia, headache, fatigue, body ache - Peripheral edema | Glucocorticoids | None |
| 19 | 71 | Do not wish to disclose | Do not wish to disclose | Vasculitis | Pfizer-BioNTech | - High grade fever - Tachycardia - Gland swelling | - Fever, chills, headache, fatigue - Peripheral edema | Methotrexate | None |
| 20 | 33 | F | Caucasian | None (HC) | Pfizer-BioNTech | - Tachycardia, Palpitations | - Injection site pain - Myalgia, fatigue, body ache, fever - Diarrhoea - Chest pain, tachycardia, palpitations - Weakness and tingling sensation in feet and legs | None | None |
| 21 | 66 | F | Caucasian | Myositis/ ASSD | Moderna | - Severe nausea, vertigo - Severe dehydration | - Injection site pain - Myalgia, fatigue, body ache, fever, joint pain | None | Asthma |
| 22 | 30 | F | Hispanic | Antiphospholipid syndrome | Pfizer-BioNTech | - Deep vein thrombosis | - Joint pain - Peripheral edema - Weakness and tingling sensation in feet and legs | None | None |
| 23 | 28 | F | Mixed | SLE | Pfizer-BioNTech | - Hypovolemic shock | - Severe diffuse body rash (hives) | Azathioprine + HCQ + Sulfasalazine | None |
| 24 | 32 | M | Caucasian | Thrombotic thrombocytopenic purpura (TTP) | Oxford/AstraZeneca | - High blood pressure | - Injection site pain - Fever - Dizziness - Visual disturbances - Bleeding/bruising on the body | None | Stroke, Leukopenia, severe chronic neutropenia |
| 25 | 59 | F | Hispanic | MCTD | Pfizer-BioNTech | - Stroke | - Injection site pain - Myalgia, fatigue, body ache, fever, joint pain, headache - Nausea/vomiting, diarrhoea - Rash - Dizziness, fainting - Visual disturbances - Bleeding/bruising on the body | None | Rheumatoid arthritis, Sjogren’s syndrome, SLE |
| 26 | 51 | M | Caucasian | Vasculitis | Moderna | - Vasculitis (poly arteritis nodosa) | - Chest pain, tachycardia, palpitations, rise in BP | Glucocorticoids | HTN |

SAID: systemic autoimmune and inflammatory disorders; HC: healthy controls; IS: immunosuppressive; F: female; M: male; SLE: systemic lupus erythematosus; MMF: mycophenolate mofetil; HCQ: hydroxychloroquine; IVIg/scIg: intravenous immunoglobulin/subcutaneous immunoglobulin; CKD: chronic kidney disease; HC: healthy control; ASSD: antisynthetase syndrome; HTN: hypertension; T2DM: type 2 diabetes mellitus; ILD: interstitial lung disease; TNF: tumour necrosis factor; OM: Overlap myositis with SLE or Sjogren’s syndrome or systemic sclerosis or rheumatoid arthritis; MCTD: mixed connective tissue disease

COVID-19 Vaccination in Autoimmune Diseases-2 (COVAD-2) Study Group Author List and Affiliations

**India**

1. **Dr Yogesh Preet Singh**- Division of Rheumatology and Clinical Immunology, Department of General Medicine, Himalayan Institute of Medical sciences, Swami Rama University, Jolly Grant, Dehradun - 248140, Uttarakhand, India
2. **Dr Rajiv Ranjan** -Clinical Immunology & Rheumatology at Columbia Asia, Palam Vihar, Gurgaon, Haryana, India
3. **Dr Avinash Jain**- Department of Clinical Immunology and Rheumatology, SMS Medical College and Hospital, Jaipur, Rajasthan.
4. **Dr Sapan C Pandya**- Clinical Immunologist and Rheumatologist, Rheumatic Disease Clinic, Vedanta Institute of Medical Sciences, Navrangpura, Ahmedabad 380009, Gujarat.
5. **Dr Rakesh Kumar Pilania**- Pediatric Allergy Immunology Unit, Department of Pediatrics, Post Graduate Institute of Medical Education and Research, Chandigarh.
6. **Dr Aman Sharma**, Professor, Clinical Immunology and Rheumatology Services, Department of Internal Medicine, Post Graduate Institute of Medical Education and Research, Chandigarh.
7. **Dr Manesh Manoj M**- Department of Clinical Immunology and Rheumatology, AKG Memorial Hospital and Dr Shenoy’s CARE (Centre for Arthritis and Rheumatism Excellence), Kannur, Kerala.
8. **Dr Vikas Gupta**, Rheumatology, Dayanand Medical College and Hospital, Ludhiana, Punjab 141001, India.
9. **Dr Chengappa G Kavadichanda**, Department of Clinical Immunology, Jawaharlal Institute of Postgraduate Medical Education and Research, Puducherry, India.
10. **Dr Pradeepta Sekhar Patro**, Department of Clinical Immunology and Rheumatology, Sunshine Hospitals, Plot No 208, Cuttack Puri Road, Laxmisagar, Bhubaneshwar, Odisha.
11. **Dr Sajal Ajmani**, Arthritis and Rheumatology clinic, New Delhi, Delhi. sajalajmani@gmail.com
12. **Dr Sanat Phatak**, Rheumatology & Immunology, Department of Rheumatology and Immunology, KEM Hospital, Pune, Maharashtra.
13. **Dr Rudra Prosad Goswami**, Department of Rheumatology, All India Institute of Medical Sciences, New Delhi, Delhi.
14. **Dr Abhra Chandra Chowdhury**, Rheumatologist, AMRI Hospital, Dhakuria, Kolkata, West Bengal.
15. **Dr Ashish Jacob Mathew**, Department of Clinical Immunology & Rheumatology, Christian Medical College and Hospital, Vellore, Tamil Nadu 632004.
16. **Dr Padnamabha Shenoy**, Dr Shenoy’s CARE (Centre for Arthritis and Rheumatism Excellence), Kannur, Kerala
17. **Dr Ajay Asranna**, Department of Neurology, NIMHANS, Bengaluru, Karnataka
18. **Dr Keerthi Talari Bommakanti**, Consultant Rheumatologist, Yashoda hospital, Behind Hari Hara Kala Bhavan, Secunderabad - 500003, T.S. Hyderabad, Telangana
19. **Dr Anuj Shukla**, Niruj Rheumatology Clinic, 209 Rajvi Complex, Rambaug, Ahmedabad, 380008, Gujarat.
20. **Dr Arunkumar R Pande-** LEDTC Clinic, Gomti Nagar, Lucknow, Uttar Pradesh, India.
21. **Prithvi Sanjeevkumar Gaur-** Smt. Kashibai Navale Medical and General Hospital, Pune, India.
22. **Dr Mahabaleshwar Mamadapur-** Department of Clinical Immunology and Rheumatology, Sanjay Gandhi Postgraduate Institute of Medical Sciences, Lucknow, India
23. **Akanksha Ghodke**- Mahatma Gandhi Mission Medical College, Navi Mumbai, Maharashtra, India
24. **Dr Kunal Chandwar**- Department of Clinical Immunology and Rheumatology, King George's Medical University, Lucknow, Uttar Pradesh, India

**Pakistan**

- - - 1. **Zoha Zahid Fazal.** Medical College, The Aga Khan University, Karachi, Pakistan

**Turkey**

1. **Dr Sinan Kardeş**- Department of Medical Ecology and Hydroclimatology, Istanbul Faculty of Medicine, Istanbul University, Capa-Fatih, 34093, Istanbul, Turkey.
2. **Dr Döndü Üsküdar Cansu**, Division of Rheumatology, Department of Internal Medicine, Eskişehir Osmangazi University, 26480, Eskişehir, Turkey.
3. **Dr Reşit Yıldırım**- Division of Rheumatology, Department of Internal Medicine, Eskişehir Osmangazi University, 26480, Eskişehir, Turkey.

**United Kingdom**

1. **Dr Armen Yuri Gasparyan-** Departments of Rheumatology and Research and Development, Dudley Group NHS Foundation Trust, Russells Hall Hospital, North Block, Clinical Research Unit, Dudley, West Midlands, DY1 2HQ, United Kingdom

**France**

1. **Dr. Margherita Giannini**, Explorations fonctionnelles musculaires, service de physiologie, Hôpitaux universitaires de Strasbourg; EA3072, fédération de médecine translationnelle.
2. **Dr François Maurier-** Service de Médecine Interne, Hôspital Robert Schuman, Rue de Champ Montoy, 57070 Vantoux, France.
3. **Dr Julien Campagne-** Service de Médecine Interne, Hôspital Robert Schuman, Rue de Champ Montoy, 57070 Vantoux, France
4. **Dr Alain Meyer-** 1. Centre National de Référence des Maladies Systémiques et Auto-immunes Rares Grand-Est Sud-Ouest (RESO), Service de humatologie, Service de physiologie, Unité d’explorations fonctionnelles musculaires, Hôpitaux Universitaires de Strasbourg, Strasbourg, France; 2. EA3072, Fédération de Médecine Translationelle, Université de Strasbourg, Strasbourg, France

**Italy**

1. **Dr. Nicoletta Del Papa**, Unità operativa complessa (UOC) Day Hospital Reumatologia via Gaetano Pini 9, Centro Specialistico Ortopedico Traumatologico, Gaetano Pini-CTO, Milano, Italy.
2. **Dr. Gianluca Sambataro**, Medico Immunologia e reumatologia presso, Artoreuma S.R.L., Cors S. Vito 53, 95030 Mascalucia, CT, Italy.
3. **Dr. Atzeni Fabiola**,  Rheumatology Unit,  University of Messina, Messina, Italy.
4. **Dr. Marcello Govoni**, Professor, Department of Medical Sciences, Complex Operative Unit and Rheumatology Unit of S.Anna University Hospital, University of Ferrara, Via A. Moro 8, 44124- Cona (FE), Italy
5. **Dr Simone Parisi**, Epidemiology Unit, Italian Society for Rheumatology, Milan, Italy; Rheumatology Unit, Azienda Ospedaliera Città della Salute e della Scienza di Torino, Torino, Italy.
6. **Dr Elena Bartoloni Bocci**, Associate Professor, Department of Medicine and Surgery, MED/16- Rheumatology, Università degli studi di Perugia, P.zza Università - 06123 – Perugia, Italy.
7. **Dr. Gian Domenico Sebastiani**, U.O.C. Reumatologia, Ospedale San Camillo-Forlanini, Roma, Italy.
8. **Dr Enrico Fusaro**, Rheumatology Unit, Azienda Ospedaliero-Universitaria Città della Salute e della Scienza di Torino, Torino, Italy.
9. **Dr Marco Sebastiani**, Rheumatology Unit, University of Modena and Reggio Emilia, Azienda Ospedaliero-Universitaria Policlinico di Modena, Via del Pozzo, 41125, Modena, Italy. marco.sebastiani@unimore.it
10. **Dr Luca Quartuccio**, Clinic of Rheumatology, Department of Medicine (DAME), ASUFC, University of Udine, Udine, Italy.
11. **Dr Franco Franceschini**, Rheumatology and Clinical Immunology Unit, Department of Clinical and Experimental Sciences, ASST Spedali Civili and University of Brescia, Italy.
12. **Dr Pier Paolo Sainaghi**, Department of Translational Medicine, Università del Piemonte Orientale UPO, Novara, Italy; Division of Internal Medicine, Immunorheumatology Unit, CAAD (Center for Translational Research on Autoimmune and Allergic Disease) Maggiore della Carità Hospital, Novara, Italy; IRCAD, Interdisciplinary Research Center of Autoimmune Diseases, Novara.
13. **Dr Giovanni Orsolini**, Department of Medicine, Rheumatology Unit, University of Verona, Verona, Italy.
14. **Dr Rossella De Angelis**, Rossella De Angelis, Rheumatology Unit, Department of Clinical and Molecular Sciences, Polytechnic University of Marche.
15. **Dr Maria Giovanna Danielli**, Clinica Medica, Dipartimento di Scienze Cliniche e Molecolari, Università Politecnica delle Marche e Azienda Ospedali Riuniti, Ancona, Italy.
16. **Dr Vincenzo Venerito**- Department of Emergency and Organ Transplantations-Rheumatology Unit, University of Bari "Aldo Moro", Bari, Italy.
17. **Dr Silvia Grignaschi**- Rheumatology Unit, Dipartimento di Medicine Interna e Terapia Medica, Università degli studi di Pavia, Pavia, Lombardy, Italy
18. **Dr. Alessandro Giollo.** Division of Rheumatology, Department of Medicine, University of Padova Hospital Trust, Padova, Italy. alessandro.giollo@unipd.it
19. **Dr Laura Andreoli**, MD, PhD, Associate Professor: 1. Rheumatology and Clinical Immunology Unit, ASST Spedali Civili and University of Brescia, 25123 Brescia, Italy. 2. Department of Clinical and Experimental Sciences, University of Brescia, 25123 Brescia, Italy.
20. **Dr Daniele Lini**: 1. Rheumatology and Clinical Immunology Unit, ASST Spedali Civili and University of Brescia, 25123 Brescia, Italy. 2. Department of Clinical and Experimental Sciences, University of Brescia, 25123 Brescia, Italy.
21. **Dr. Alessia Alluno:** Rheumatology Unit, University of Perugia, Perugia, Italy.
22. **Dr.** **Florenzo Iannone**: DETO-Department of Emergency and Organ Transplantation-Rheumatology Unit, University of Bari, Bari, Italy
23. **Dr**. **Marco Fornaro**: DETO-Department of Emergency and Organ Transplantation-Rheumatology Unit, University of Bari, Bari, Italy

**Philippines**

1. **Dr Lisa S Traboco** Department of Medicine, Section of Rheumatology, St. Luke's Medical Center-Global City, Taguig, Philippines

**Indonesia**

**Dr Suryo Anggoro Kusumo Wibowo**, Division of Rheumatology, Department of Internal Medicine, Faculty of Medicine, Universitas Indonesia/ Dr Cipto Mangunkusumo General Hospital, Jakarta, Indonesia.

**Spain**

1. **Dr Jesús Loarce-Martos**, Rheumatology Department, Hospital Universitario Ramón y Cajal, Carretera de Colmenar Viejo, 9, 1 km, 28043, Madrid, Spain.
2. **Dr Sergio Prieto-González**, Department of Internal Medicine, Hospital Clinic of Barcelona, University of Barcelona, Barcelona, Spain.
3. **Dr Raquel Aranega,** Systemic Autoimmune Diseases Unit, Vall d'Hebron General Hospital, Medicine Dept, Universitat Autónoma de Barcelona, Barcelona, Spain.

**Japan**

1. **Dr Akira Yoshida-** Department of Allergy and Rheumatology, Nippon Medical School Graduate School of Medicine, 1-1-5 Sendagi, Bunkyo-ku, Tokyo 113-8602, Japan
2. **Dr Ran Nakashima**, Department of Rheumatology and Clinical Immunology, Graduate School of Medicine, Kyoto University, 54 Shogoin-Kawahara-cho, Sakyo-ku, Kyoto 606-8507, Japan.
3. **Dr Shinji Sato**, Division of Rheumatology, Department of Internal Medicine, Tokai University School of Medicine, 143 Shimokasuya, Isehara, 259-1193, Japan.
4. **Dr Naoki Kimura**, Department of Lifetime Clinical Immunology, Graduate School of Medical and Dental Sciences, Tokyo Medical and Dental University (TMDU), Tokyo, Japan.
5. **Dr Yuko Kaneko**, Division of Rheumatology, Department of Internal Medicine, Keio University School of Medicine, Tokyo, Japan.
6. **Takahisa Gono-** Department of Allergy and Rheumatology, Nippon Medical School Graduate School of Medicine, 1-1-5 Sendagi, Bunkyo-ku, Tokyo 113-8602, Japan

**Germany**

1. **Dr Stylianos Tomaras**- Department of Rheumatology, Helios Clinic Vogelsang-Gommern, 39245 Gommern, Germany.
2. **Dr Fabian Nikolai Proft**- Department of Gastroenterology, Infectiology and Rheumatology (including Nutrition Medicine), Charité - Universitätsmedizin Berlin, corporate member of Freie Universität Berlin and Humboldt- Universität zu Berlin, Berlin, Germany.
3. **Dr Marie-Therese Holzer**: 1. Department of Pediatrics, Pediatric Rheumatology/Special Immunology, University Hospital Wuerzburg, Josef-Schneider-Str. 2, 97080, Wuerzburg, Germany. 2. Department of Internal Medicine III. (Nephrology and Rheumatology With Section Endocrinology), University Hospital Hamburg- Eppendorf, University Hospital Hamburg-Eppendorf, Martinistraße 52, 20246, Hamburg, Germany.



**Russian Federation**

1. **Dr Margarita Aleksandrovna Gromova**, Pirogov Russian National Research Medical University (RNRMU), Moscow, Russian Federation.

**Israel**

1. **Mr Or Aharonov**, Department of Gerontology, Faculty of Social Welfare and Health Science, University of Haifa, Haifa, Israel.

**Hungary**

1. **Dr Melinda Nagy-Vincze**- 1. Division of Clinical Immunology, Faculty of Medicine, University of Debrecen, Móricz Zsigmond út 22, Debrecen, H-4032, Hungary. 2. Gyula Petrányi Doctoral School of Clinical Immunology and Allergology, University of Debrecen, Debrecen, Hungary.
2. **Dr Zoltán Griger**: 1. Division of Clinical Immunology, Faculty of Medicine, University of Debrecen, Móricz Zsigmond út 22, Debrecen, H-4032, Hungary.

**Denmark**

- - - 1. **Karen Schreiber**: 1. Danish Hospital for Rheumatic Diseases, 6400 Sønderborg, Denmark. 2.
         Department of Regional Health Research (IRS), University of Southern Denmark, 5230 Odense, Denmark. 3. Thrombosis and Haemostasis, Guys and St Thomas’ NHS Foundation Trust, London SE1 7EH, UK

**Morocco**

1. **Dr Ihsane Hmamouchi-** Professor (Associate), Laboratoire d'épidémiologie et de recherche clinique, La Faculté de Médecine et de Pharmacie de Rabat, Morocco.
2. **Dr Pr Imane El bouchti**, Head of the Rheumatology Department, Mohammed VI University Hospital, Marrakech, Morocco.
3. **Dr. Zineb Baba**: Department of Rheumatology, Mohammed VI University Hospital, Marrakech, Morocco.

**Nigeria**

1. **Dr Uyi Ima-Edomwonyi-** Consultant, Department of Internal Medicine, Lagos University Teaching Hospital, Lagos, Nigeria.
2. **Dr Ibukunoluwa Dedeke**- Department of Medicine, University College Hospital Ibadan, Ibadan, Nigeria.
3. **Dr Emorinken Airenakho**- Consultant Rheumatologist, Irrua Specialist Teaching Hospital, KM 87 Benin Auchi Rd, 310115, Irrua, Nigeria.
4. **Dr Nwankwo Henry Madu**- Lecturer 1, Department of Medicine, Nnamdi Azikiwe University, Awka.
5. **Dr Abubakar Yerima**- Department of Medicine, University of Maiduguri Teaching Hospital, Maiduguri, Borno State, Nigeria.
6. **Dr Hakeem Olaosebikan**- Consultant Rheumatologist, Lagos State University Teaching Hospital/ Lagos State University College of Medicine,Ikeja, Lagos, Nigeria. +2348035751154.

**Ethiopia**

- - - 1. **Dr Becky A.** Rheumatology Unit, Internal Medicine Department, Addis Ababa University, Addis Ababa, Ethiopia.

**Mauritius**

**Dr Ouma Devi Koussougbo**. Rheumatology, Victoria hospital, Mauritius.

**Mozambique**

**Dr Elisa Palalane**: Rheumatology and Internal Medicine, Hospital Central de Maputo, Maputo, Mozambique

**Australia**

1. **Dr Daman Langguth**- Department of Immunology, Sullivan Nicolaides Pathology, Brisbane, Queensland, Australia.
2. **Dr Vidya Limaye**- Consultant Rheumatologist, Royal Adelaide Hospital, Associate Professor of Rheumatology, Discipline of Medicine, University of Adelaide, Australia.
3. **Dr Merrilee Needham**- 1. Neurology Department, Fiona Stanley Hospital, Murdoch, Australia; 2. Institute for Immunology and Infectious Diseases, Murdoch University, Murdoch, Australia; 3. Perron Institute for Neurological and Translational Science, Nedlands, Australia; 4. University of Notre Dame, Fremantle, Australia.
4. **Dr Nilesh Srivastav**- Alfred Health, The Alfred, Caulfield Hospital, Sandringham Hospital, Melbourne, Victoria, Australia.

**Canada**

1. **Dr Marie Hudson-** Department of Medicine, McGill University, Montreal, Quebec; Division of Rheumatology, Jewish General Hospital, Montreal, Quebec, and Lady Davis Institute, Jewish General Hospital, Montreal, Quebec, Canada.
2. **Dr Océane Landon-Cardinal-** Department of Medicine, University of Montreal, Montreal, Canada. Department of Medicine, CHUM Research Centre, Montreal, Canada. Department of Internal Medicine and Clinical Immunology and Inflammation-Immunopathology-Biotherapy Department (I2B), Pitié-Salpêtrière University Hospital, AP-HP, East Paris Neuromuscular Diseases Reference Center, Inserm U974, Sorbonne Université, Paris, France; Center of Reference for Neuromuscular Disorders AOC, Department of Neurology, Bordeaux University Hospital, Bordeaux, France; Polyvalent and Oncologic Radiology Department, Musculoskeletal Unit, Pitié-Salpêtrière University Hospital, AP-HP, Paris, France. o.landoncardinal@gmail.com.

**Colombia**

- - - 1. **Dr Wilmer Gerardo Rojas Zuleta**- Department of Rheumatology, Universidad de Antioquia, Cl. 67 #53 - 108, Medellín, Colombia.
      2. **Dr. Álvaro Arbeláez: Médico especialista en Reumatología y Medicina Interna. Universidad Libre. Clínica Imbanaco. Clínica de Artritis Temprana. Cali, Valle, Colombia.**
      3. **Dr. Javier Cajas:** Institute of Rheumatology Ferdinand Chalem, Bogota, Colombia.

**Portugal**

1. **Dr José António Pereira Silva**- Rheumatology Department, Centro Hospitalar e Universitário de Coimbra EPE, and Coimbra Institute of Clinical and Biomedical Research (iCBR), Faculty of Medicine, University of Coimbra, Portugal.

2. **Dr João Eurico Fonseca**- Hospital de Santa Maria, Centro Hospitalar Lisboa Norte Centro Académico de Medicina de Lisboa, Lisboa, Portugal; Instituto de Medicina Molecular, Faculdade de Medicina, Universidade de Lisboa, Lisboa, Portugal.

**Ukraine**

**Dr Olena Zimba-** Department of Internal Medicine #2, Danylo Halytsky Lviv National Medical University, Lviv, Ukraine.

**Hong Kong**

1. **Dr Ho So**- Assistant Professor, Department of Medicine & Therapeutics, Faculty of Medicine, The Chinese University of Hong Kong.

**Peru**

1. **Dr Manuel Francisco Ugarte-Gil**- Servicio de Reumatología, Hospital Nacional Guillermo Almenara Irigoyen, EsSalud, Lima, Peru. School of Medicine, Universidad Científica del Sur, Lima, Peru.
2. **Dr. Lyn Chinchay**: Seguro Social de Salud del Peru (ESSALUD), Lima, Peru
3. **Dr. José Proaño Bernaola**: 1. Cayetano Heredia National Hospital, Lima, Peru. 2. Anglo-American Clinic. San Isidro, Lima, Peru. 3. Peruvian University Cayetano Heredia, Lima, Peru. 4. Rheumatologist, San Judas Tadeo Clinic - Research Center (ENDOMED), Lima. 5. Environmental and Food Health - DIRIS – Lima, Peru
4. **Dr. Victorio Pimentel**: Rheumatology Department, Hospital Guillermo Almenara Irigoyen, EsSalud, Av. Grau 800, La Victoria, Lima 13, Lima, Peru.

**Egypt**

1. **Dr Hanan Mohamed Fathi**- Rheumatology and Autoimmune Diseases, Faculty of Medicine, Fayoum University, Faiyum, Egypt.
2. **Dr Reem Hamdy A Mohammed**: Department of Rheumatology and Clinical Immunology, Kasr Alainy School of Medicine- Cairo University, Cairo, Egypt.

**United Arab Emirates**

1. **Dr Ghita Harifi**- Department of Rheumatology, Mediclinic Parkview Hospital, 3 Umm Suqeim St - Al Barsha Al Barsha South, Dubai, United Arab Emirates.

**Venezuela**

1. **Dr. Yurilís Fuentes-Silva**: 1. Health Sciences School, University of Oriente– Bolivar Nucleus, Ciudad Bolivar, Venezuela. 2. Centro Clínico Universitario de Oriente, Ciudad Bolivar, Venezuela.

**Paraguay**

1. **Dr. Karoll Cabriza**: Hospital de Clínicas, San Lorenzo, Paraguay.
2. **Dr.** **Jonathan Losanto**: Hospital de Clínicas, San Lorenzo, Paraguay.
3. **Dr. Nelly Colaman**: Hospital de Clínicas, San Lorenzo, Paraguay.

**Panama**

1. **Dr.** **Antonio Cachafeiro-Vilar**: Pacífica Salud-Hospital Punta Pacífica, Ciudad de Panamá, Panamá.
2. **Dr. Generoso Guerra Bautista**: Centro de Investigación Marbella, Paitilla Panamá, Panamá.
3. **Dr. Enrique Julio Giraldo Ho**: Rheumatologist, Universidad de Panamá, República de Panamá.

**Chile**

1. **Dr. Lilith Stange Nunez**: Rheumatologist, University of Valparaiso, Pontifical Catholic University of Chile.
2. **Dr. Cristian Vergara M**: Departamento de Medicina Interna, Escuela de Medicina Dirección de PostGrado y Post Título, Facultad de Medicina, Universidad de Valparaíso, Santiago, Chile.

**Dominican Republic**

1. **Dr. Jossiell Then Báez**: MD, Hospital Metropolitano de Santiago (HOMS), Santiago, Dominican Republic.

**Honduras**

1. **Dr. Hugo Alonzo**: Jefe del Departamento de Medicina Interna en Hospital de Especialidades del Seguro Social en Tegucigalpa, Honduras.
2. **Dr. Carlos Benito Santiago Pastelin**: Médico especialista, Instituto Hondureño del Seguro Social, Honduras.

**Argentina**

1. **Dr. Rodrigo García Salinas**: Rheumatology Unit, La Plata Italian Hospital, Buenos Aires, Argentina.

**Guatemala**

1. **Dr. Alejandro Quiñónez Obiols**: MD, PhD, Universidad Mariano Gálvez de Guatemala, Guatemala City, Guatemala.
2. **Dr. Nilmo Chávez**: MD, Instituto Guatemalteco de Seguridad Social - Universidad San Carlos de Guatemala, Ciudad de Guatemala, Guatemala.
3. **Dr. Andrea Bran Ordóñez**: Hospital El Pilar, Universitario Esperanza, Guatemala City, Guatemala

**Cuba**

1. **Dr. Gil Alberto Reyes Llerena**: Surgical Medical Research Center (CIMEQ), Rheumatology Service, 216th Street and 11B, Siboney. Beach. Havana, Cuba. Phone: 209-1431.

**Puerto Rico**

1. **Dr. Radames Sierra-Zorita**: University of Puerto Rico, School of Medicine, San Juan, Puerto Rico.

**Costa Rica**

1. **Dr. Dina Arrieta**: Hospital México, Caja Costarricense del Seguro Social, San José de Costa Rica, Costa Rica.
2. **Dr. Eduardo Romero Hidalgo**: Jackson's Memorial Medical Center, San Ramon, Alajuela, Costa Rica.
3. **Dr. Ricardo Saenz**: Jefe Servicio Reumatología Hospital Dr. Calderón Guardia C.C.S.S., Costa Rica

**Nicaragua**

1. **Dr. Idania Escalante M**.: 1. Internal Medicine, Rheumatologist, Oscar Danilo Rosales School Hospital. Leon-Nicaragua. 2. Medicine professor of National Autonomous University of Nicaragua. idaescmen@gmail.com

**Ecuador**

1. **Dr. Wendy Calapaqui**: Instituto Ecuatoriano de Seguridad Social. Centro de atención ambulatoria “El Batán”, Quito, Ecuador.
2. **Dr. Ivonne Quezada**: Hospital de Especialidades Eugenio Espejo, Quito, Ecuador.

**Bolivia**

1. **Dr. Gabriela Arredondo:** Department of Rheumatology, Medical Center, Santa Cruz, Bolivia; Department of Rheumatology, Alemana Clinic, La Paz, Bolivia
